# Sishen Pill treats spleen-kidney yang deficiency IBS-D by influencing ATPase activity and intestinal microbiota

**DOI:** 10.3389/fmicb.2025.1610928

**Published:** 2025-11-12

**Authors:** Qi Long, Liwen Li, Zhoujin Tan, Na Deng

**Affiliations:** 1 School of Traditional Chinese Medicine, Hunan University of Chinese Medicine, Changsha, China; 2 Hunan Key Laboratory of Traditional Chinese Medicine Prescription and Syndromes Translational Medicine, Changsha, China

**Keywords:** Sishen Pill, intestinal microbiota, spleen-kidney yang deficiency IBS-D, ATPase activity, intestinal digestive enzyme activity

## Abstract

**Background:**

Sishen Pill (SSP) has therapeutic effects on spleen-kidney yang deficiency diarrhea-predominant irritable bowel syndrome (IBS-D). Studies have shown that IBS-D with spleen-kidney yang deficiency is characterized by intestinal microbiota dysbiosis and impaired ATPase activity. After SSP treatment, the abundance of beneficial microbiota increased, the balance of the intestinal microbiota was restored, and ATPase activity improved, suggesting that SSP may exert its therapeutic effects by modulating the intestinal microbiota and restoring ATPase activity.

**Methods:**

Forty SPF-grade female mice were randomly allocated into four groups (n = 10 per group): normal group (CC), spontaneous recovery group (MC), pinaverium bromide treatment group (PBT), and SSP treatment group (SSP). The IBS-D mouse model was successfully established by adenine and *Folium sennae* combined with restraint-tail clamping stress. Following model validation, the animals received SSP aqueous extract treatment. Subsequent evaluations included: (1) assessment of behavioral parameters according to diagnostic criteria; (2) quantification of Na^+^-K^+^-ATPase and Ca^2+^-Mg^2+^-ATPase activities via ELISA; (3) measurement of intestinal digestive enzyme activity and intestinal microbial activity;(4) characterization of the intestinal microbiota composition via 16S rRNA gene sequencing analysis.

**Results:**

(1) Compared with CC group, SSP group presented significantly increased Na^+^-K^+^-ATPase and Ca^2+^-Mg^2+^-ATPase activities (*p* < 0.05). (2) Enzyme activity assays revealed that digestive enzyme activities were significantly increased in SSP group, with marked increases in lactase and amylase (*p* < 0.01) and a moderate increase in sucrase. (3) Intestinal microbial activity was notably increased in SSP group (*p* < 0.01). (4) Correlation analysis showed *Clostridioides* had a strong positive correlation with lactase (*p* < 0.01) and positive links to amylase and microbial activity (*p* < 0.05). *Desulfovibrio* demonstrated significant negative correlations with microbial activity (*p* < 0.01). In the comparison between PBT group and SSP group, Ca^2+^-Mg^2+^-ATPase showed a positive correlation with *Maribacter* and *Scatolibacter* (*p* < 0.05). Amylase exhibited a significant negative correlation with *Scatolibacter* (*p* < 0.01). Fecal microbial activity was negatively correlated with *Scatolibacter* (*p* < 0.01).

**Conclusion:**

SSP significantly alleviates symptoms of spleen-kidney yang deficiency in IBS-D by regulating intestinal microbiota, increasing Na^+^-K^+^-ATPase and Ca^2+^-Mg^2+^-ATPase activity, and enhancing lactase, amylase and sucrase activity and intestinal microbial activity.

## 1 Introduction

Irritable bowel syndrome (IBS) is a common functional gastrointestinal disorder characterized by recurrent abdominal pain and abnormal bowel movements. Based on the type of bowel movements, IBS can be classified into diarrhea-predominant (IBS-D), constipation-predominant (IBS-C), mixed (IBS-M), and unclassified (IBS-U) types on the basis of the type of bowel movement (Bian et al., 2017). Among these subtypes, IBS-D is the most common and is often accompanied by gastrointestinal inflammation, intestinal microbiota dysbiosis, and dysfunction of the intestinal barrier (Kamiya et al., 2020). Modern medical research has shown that an imbalance in intestinal microbiota plays a key role in the pathogenesis of IBS-D. Pathogenic bacteria in the intestine can increase mucosal permeability, while probiotics can strengthen the intestinal mucosal barrier (Liu et al., 2021). IBS patients often exhibit small intestinal bacterial overgrowth (SIBO) (Sachdeva et al., 2011), and short-chain fatty acids (SCFAs) are crucial for maintaining intestinal health, energy metabolism, and immune regulation. Studies have shown that a deficiency in SCFAs, particularly butyrate, may impair the synthesis of acetyl-CoA via fatty acid β-oxidation, leading to decreased ATP production (Zhang et al., 2018). This reduction in ATP production may decrease ATPase activity, further exacerbating intestinal dysfunction. The impairment of ATP generation results in an insufficient energy supply to colonic cells, contributing to symptoms such as diarrhea (Zhang et al., 2019).

Traditional Chinese medicine (TCM) views IBS-D as a type of “diarrhea,” often involving spleen and kidneys, leading to a deficiency in spleen and kidney yang. Traditional treatments often use methods such as “supplementing fire to generate earth,” which aims to warm and tonify spleen and kidneys while astringing the intestines to stop diarrhea. In recent years, SSP, a classical Chinese herbal formula composed of six ingredients—*Psoralea corylifolia*, *Myristica fragrans*, *Schisandra chinensis*, *Evodia rutaecarpa*, *Zingiber officinale* and *Ziziphus jujuba*—has shown significant therapeutic effects in the treatment of IBS-D (Zhang et al., 2021; Chen et al., 2020). In the SSP formula, *P. corylifolia* is used as the monarch herb to warm and supplement the fire of the mingmen (life gate); *M. fragrans* serves as the minister herb to warm the spleen and stomach and astreses the intestines to stop diarrhea; it is supplemented with *E. rutaecarpa* to disperse the internal cold; it is assisted by *S. chinensis* to astringe and warm, *Z. officinale* to warm the stomach and dispel cold, and *Z. jujuba* to tonify the spleen and stomach. Together, these herbs work synergistically to warm the kidneys, tonify the spleen, and astringe the intestines to stop diarrhea. Research suggests that SSP can regulate intestinal microbiota balance and restore intestine health. Specifically, it reduces the abundance of harmful bacteria (such as Proteobacteria and *Mycoplasma*) and increases the abundance of beneficial bacteria (such as *Clostridium* and *Lactobacillus*), improving intestinal barrier function and energy metabolism (Wang et al., 2024). Furthermore, SSP has been shown to regulate intestinal metabolites, such as short-chain fatty acids (SCFAs), thereby alleviating symptoms of diarrhea and reducing colon mucosal inflammation in patients with IBS-D (Zhang et al., 2025). In addition, SSP enhances intestinal energy metabolism by modulating the activity of key enzymes, including Na^+^-K^+^-ATPase and Ca^2+^-Mg^2+^-ATPase. This regulation helps restore normal epithelial cell function. Studies have demonstrated that SSP increases ATP levels, restores ATPase activity, and promotes energy supply to colonic cells, ultimately improving the clinical manifestations of IBS-D (Ge et al., 2022). Given these effects, SSP holds potential as a therapeutic strategy for IBS-D associated with spleen-kidney yang deficiency, by regulating both the intestinal microbiota and energy metabolism.

This study aims to investigate the clinical effects of SSP in treating IBS-D associated with spleen-kidney yang deficiency, focusing on its ability to regulate intestinal microbiota, restore the activity of Na^+^-K^+^-ATPase and Ca^2+^-Mg^2+^-ATPase, and provide novel therapeutic strategies and drug targets for IBS-D. To achieve this, we developed a mouse model of spleen-kidney yang deficiency IBS-D by a combination of adenine, *Folium senna*, and restraint tail-clamping techniques. We then examined the effects of SSP treatment on the small intestine microbiota, ATPase activity, and digestive enzyme function. Through this mechanistic approach, we aim to offer new insights and identify potential drug targets for the treatment of IBS-D.

## 2 Materials and methods

### 2.1 Animals

Forty female SPF-grade Kunming mice (18–22 g) were purchased from Hunan Slack Jingda Laboratory Animal Co., Ltd. (animal license number: SCXK (Xiang) 2019–0004). To minimize the impact of sex on intestinal microbiota, only female mice were used (Wu et al., 2022; Fang et al., 2025). The mice were housed at the Laboratory Animal Center of Hunan University of Chinese Medicine under controlled conditions: 23–25 °C, 50–70% humidity, a 12-h light/dark cycle, and ad libitum access to food and water. All procedures followed the ethical guidelines approved by the Ethics Committee of the Laboratory Animal Center (approval number: HNUCM21-2404-26). The feed was supplied by Beijing Huafukang Biotechnology Co., Ltd. (Beijing Feed Certificate: (2019) 06076).

### 2.2 Experimental drugs and reagents

#### 2.2.1 Experimental drugs

Adenine was prepared at a concentration of 50 mg/kg in sterile water (Changsha Yaer Bio-Tech Co., Ltd., EZ7890C450). To prepare a 5 mg/mL suspension, 20 mg of adenine was dissolved in 0.4 mL of sterile water at 0 °C. Pinaverium bromide tablets (SUEZ Pharmaceutical Company, France, HJ20160396) were crushed, sieved, dissolved in distilled water to a concentration of 2.163 g/mL, and stored at 4 °C.

*Folium sennae* (Anhui Shenghaitang Traditional Chinese Medicine Decoction Pieces Co., Ltd., batch number: 20190605611) were soaked in boiling water, filtered, and concentrated to 1 g/mL via a rotary evaporator to prepare an aqueous decoction (Li et al., 2023). The SSP decoction was composed of *P. corylifolia* (231001), *M. fragrans* (23083107), *S. chinensis* (231201), *E. rutaecarpa* (230801), *Z. officinale*, and *Z. jujuba* (2301001) in a 4:2:2:1:2:2 ratio, totaling 39 g. These herbs were sourced from the Pharmacy of the First Affiliated Hospital of Hunan University of Chinese Medicine. For preparation, the herbs were soaked in 300 mL of water for 30 min, then boiled and simmered for an additional 30 min before filtering. The herbal residues were then boiled in 200 mL of water using the same method. The two extracts were combined and concentrated to a final concentration of 1 g/mL (Xie et al., 2024a).

#### 2.2.2 Reagents and kits

FDA (fluorescein diacetate, Shanghai Yuanye Biotechnology Co., Ltd.) was mixed with acetone (Hunan Huihong Reagent Co., Ltd.) at a ratio of 2:1 to prepare the FDA stock solution. The FDA stock solution was then diluted with PBS to a final concentration of 10 μg/mL. ONPG reagent (o-nitrophenyl β-d-galactopyranoside, Shanghai Yuanye Biotechnology Co., Ltd.) and DNS reagent (3,5-dinitrosalicylic acid, Shanghai Runcheng Biotechnology Co., Ltd.) were used in the experiments. The Na^+^-K^+^-ATPase assay kit (Lot No. JM-11845 M2) and Ca^2+^-Mg^2+^-ATPase assay kit (Lot No. JM-12156M2) were purchased from Jiangsu Jingmei Biotechnology Co., Ltd.

## 3 Methods

### 3.1 Grouping and modeling methods

After 3 days of adaptive feeding, 40 mice were randomly divided into two groups: a normal group (CC; 10 mice) and a model group (MM; 30 mice). The IBS-D mouse model with spleen-kidney yang deficiency was induced by administering adenine + *Folium sennae* decoction combined with restraint-tail clamping stress, as described in a previous study (Deng et al., 2024). One week prior to modeling, the model group was gavaged with an adenine suspension [50 mg/(kg·d), 0.4 ml per mouse] for 14 consecutive days. Beginning on the 8th day of modeling, the limbs of the model group mice were restrained in centrifuge tubes, and the distal third of their tails were clamped for 1 h daily for 7 days. Starting from day 10, model group mice were gavaged with *Folium sennae* decoction in the afternoon [10 g/(kg·d), 0.4 ml/mouse, once a day, for 5 consecutive days], while CC received sterile water at the same dosage.

**Figure 1:**
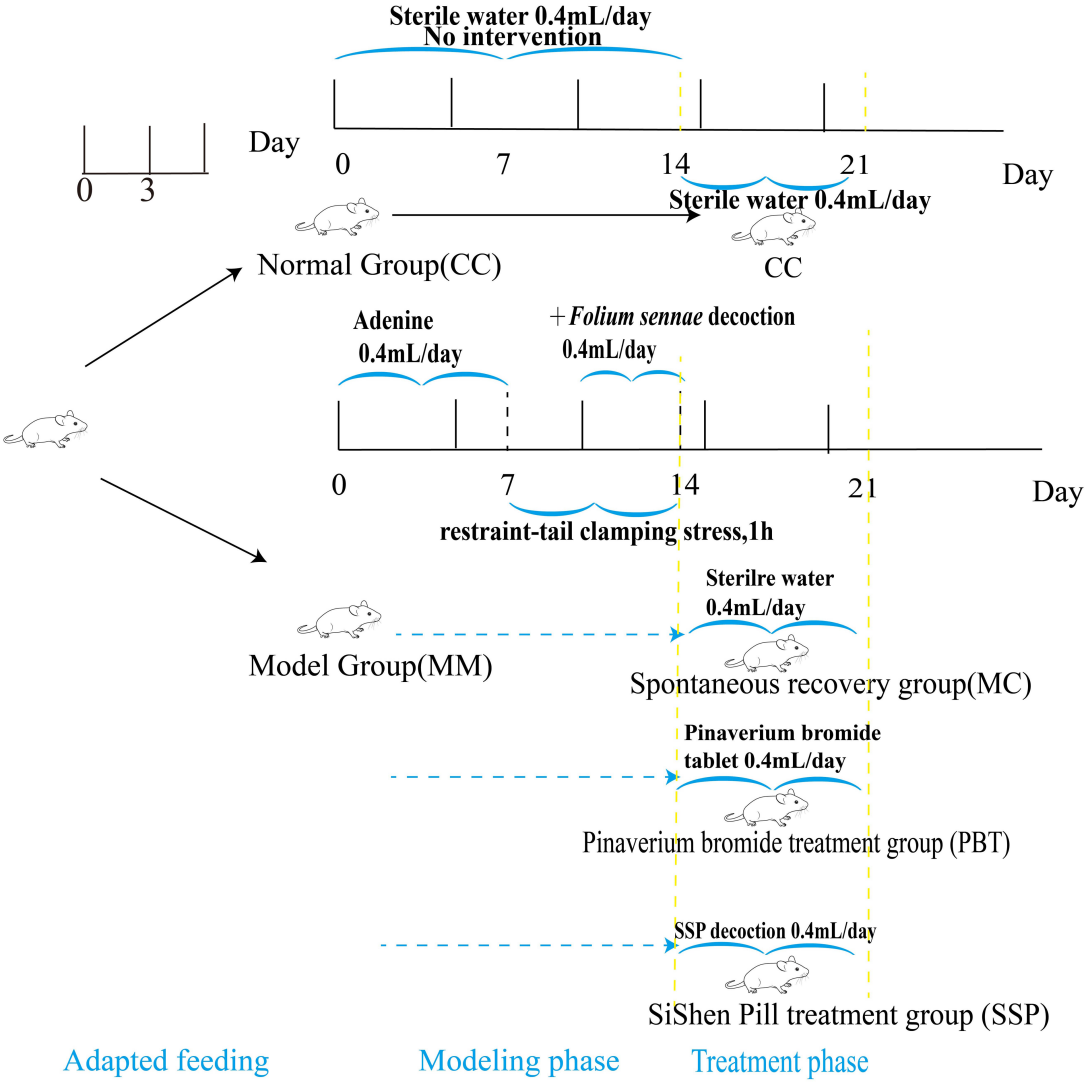
Experimental design and general conditions of the animals. CC, normal group; MC, spontaneous recovery group; PBT, pinaverium bromide tablet group; SSP, Sishen Pill group.

Once the model was established, the model group was randomly divided into three treatment groups: MC, PBT, and SSP group, with 10 mice in each group. The pinaverium bromide tablet group received a suspension at a clinically equivalent dose (21.63 mg/kg), 0.4 mL/mouse, twice daily for 7 days. The SSP group was gavaged with a decoction [5 g/(kg·d), 0.4 mL/mouse, twice daily for 7 days]. The normal and spontaneous recovery groups were gavaged with sterile water at the same dosage and frequency (Figure 1).

### 3.2 Model evaluation criteria

On the basis of the diagnostic criteria outlined in the “2023 Expert Consensus on TCM Diagnosis and Treatment of Diarrhea” and the “2024 Expert Consensus on TCM Diagnosis and Treatment of Irritable Bowel Syndrome,” we established evaluation indices for spleen-kidney yang deficiency IBS-D. Clinically, this condition is characterized by recurrent abdominal pain, bloating, and discomfort (primary symptoms), accompanied by cold intolerance, a preference for warmth or pressure, and lumbar/knee weakness (secondary symptoms). The diagnosis is made when both primary and secondary symptoms are present. To correlate clinical manifestations with measurable parameters, we observed general behavior (activity level and lethargy) as indicators of “fatigue and sluggishness”; measured fecal water content to assess “dawn diarrhea with undigested food”; and monitored anal temperature, huddling, and arched-back behavior to reflect “cold limbs.” Additionally, body weight change, food intake, and water intake were used to represent “poor appetite and emaciation.”

### 3.3 General conditions

The normal group of mice was used as a control, and the general condition of the mice before and after modeling, including body weight, rectal temperature, food intake, water intake, open field test results, activity levels, mental state, and fecal condition, was observed.

### 3.4 Determination of the weight change rate and fecal moisture content

The mice were weighed, and feces were collected at the end of the adaptation period (Day 1 of modeling), Day 14 of modeling, and Day 21 of treatment. The wet weight of the fecal samples were recorded. The samples were then dried at 110 °C until they reached a constant weight, after which the dry weight was recorded to calculate the fecal water content. The formula for calculating the body weight change rate is as follows:

Modeling phase: Body weight change rate (%) = (Body weight on Day 14) – (Body weight on Day 1 of the modeling phase)/(Body weight on Day 1 of the modeling phase) × 100%.

Treatment phase: Body weight change rate (%) = (Body weight on Day 21) – (Body weight on Day 14)/(Body weight on Day 14) × 100%.

Fecal water content (%) = (Fecal wet weight) – (Fecal dry weight)/(Fecal wet weight) × 100% (Qiao et al., 2023a).

### 3.5 Open field test

The open field test (OFT), reflects the emotional state of a mouse and is a classic method for assessing motor function and anxiety. On day 21 at the end of the treatment, five mice from each group were selected randomly for testing in the KSYY-OP-V4.0 Mouse Open Field Real-Time Detection and Analysis System. Their movement distance and average speed within 5 min were recorded. After each test, feces and urine were removed, and the box was wiped with 75% ethanol. The procedure was repeated five times, and average values were calculated (Qiao et al., 2023b).

### 3.6 Measurement of Na^+^-K^+^-ATPase and Ca^2+^-Mg^2+^-ATPase in serum

Whole blood was collected on day 21 for serum analysis, and the activities of Na^+^-K^+^-ATPase and Ca^2+^-Mg^2+^-ATPase in serum were measured via ELISA assay. The specific procedure involved collecting whole blood samples from mice, followed by centrifugation at 3,000 revolutions per minute for 10 min to separate the serum. The plate layout, sample addition, enzyme addition, incubation, washing, color development, and reaction termination were performed according to the guidelines provided in the ELISA kit. The OD value was measured at a wavelength of 450 nm via a microplate reader (Zhu et al., 2022).

### 3.7 Collection of organs and calculation of organ indices

After blood collection on day 21, the mice were euthanized by cervical dislocation, and the spleen and thymus were completely excised for organ index calculation. The organ index, defined as the percentage of organ weight relative to body weight, serves as an indicator of organ development and health status to some extent. Organ index = Organ weight (g)/body weight (g) × 100% (Li et al., 2022).

### 3.8 Measurement of intestinal enzyme activity

On day 21, after euthanizing the mice by cervical dislocation, small intestinal contents were collected for measurement of intestinal enzyme activity. The samples were subsequently placed on a clean bench, and intestinal content samples from the jejunum to the ileum were aseptically collected via sterile forceps. After collection, each group of samples was placed in a sterile centrifuge tube containing an appropriate amount of sterile water and 7–8 glass beads, with a ratio of 3 g of contents to 50 mL of sterile water. The samples were vortexed for 2 min to ensure the complete release of enzyme-related substances. The mixture was then centrifuged at 3000 rpm at 4 °C for 10 min, and the resulting mixture was collected as the crude enzyme mixture. The activity of amylase was measured via the DNS colorimetric method. The activity of sucrase was determined at a wavelength of 540 nm, and the activity of lactase was measured via the ONPG method at a wavelength of 420 nm (Wu et al., 2021).

### 3.9 Measurement of intestinal microbial activity

Intestinal content samples were collected from the jejunum to the ileum and diluted with sterile water according to method 3.8. The mixture was then centrifuged at 3,000 rpm for 10 min to collect the supernatant, which served as the crude enzyme solution. The FDA stock solution was prepared by dissolving 2 mg of FDA in 1 mL of acetone to make a 2 mg/mL concentration. This stock solution was stored in the dark at −20 °C. A reaction solution (A solution) was prepared by adding the FDA stock solution to phosphate-buffered saline (PBS) to achieve a final concentration of 10 μg/mL of FDA. In the test group, 2 mL of the A solution was added to a tube, followed by 10 μL of crude enzyme solution. The mixture was then shaken and incubated at 24 °C for 90 min. After incubation, the reaction was terminated by adding 2 mL of acetone. For the control group, the same procedure was followed, except that acetone was added along with the A solution and crude enzyme solution before shaking. After the reaction, microbial activity was measured using a UV–Vis spectrophotometer at a wavelength of 490 nm (Xie et al., 2024b).

### 3.10 16S rRNA high-throughput sequencing of the microbiota of small intestinal contents

(1) DNA extraction: Total genomic DNA was extracted from small intestinal contents using a bacterial DNA extraction kit. DNA quantity and quality were assessed via NanoDrop spectrophotometry and agarose gel electrophoresis.

(2) PCR amplification: PCR amplification was performed using specific primers targeting the V3 + V4 region of bacterial 16S rRNA. The forward primer sequence 338F (5′-ACTCCTACGGGAGGCAGCA-3′) and the reverse primer sequence 806R (5′-GGACTACHVGGGTWTCTAAT-3′) were used.

(3) PCR product recovery and purification: PCR products were detected using 2% agarose gel electrophoresis, and purification was carried out using the Axygen^®^ AxyPrep DNA Gel Extraction Kit.

(4) Fluorescent quantification of PCR products: The Quant-it PicoGreen dsDNA Assay Kit was used to quantitate the recovered PCR amplification products. Samples were mixed in proportion according to the sequencing volume requirements for each sample.

(5) Sequencing: Library construction was performed using the Illumina TruSeq Nano DNA LT Library Prep Kit, and sequencing was conducted using the Illumina NovaSeq 6,000 platform with the NovaSeq 6,000 SP Reagent Kit v1.5 (500 cycles) (Illumina, San Diego, CA, United States) in a 2*250 bp sequencing mode. Sequencing was conducted by Shanghai Personal Biotechnology Co., Ltd. The sequencing data of the cecal content microbiota have been deposited in the NCBI database under the accession number: PRJNA1177239.

### 3.11 Bioinformatics analysis

(1) *α* Diversity analysis at the ASV level via QIIME2: α diversity indices, including the Chao1 richness estimatior, observed species, Shannon diversity index, and Simpson index, were calculated at the ASV level. Additionally, rank abundance curves were generated to visualize the richness and evenness of the ASVs (Qiao et al., 2023a).

(2) β Diversity analysis via Bray–Curtis distance: β diversity analysis was assessed via Bray–Curtis dissimilarity (Bray and Curtis, 1957). The structural differences in microbial communities across samples were visualized via principal coordinate analysis (PCoA) and nonmetric multidimensional scaling (NMDS) (Li et al., 2022).

(3) Venn diagram and LEfSe analysis: Venn diagrams were generated via the R package “Venn diagram” to show the shared and unique ASVs between samples or groups, on the basis of the presence/absence of ASVs rather than their relative abundance. Additionally, linear discriminant analysis effect size (LEfSe) was employed to identify differentially abundant taxa between groups (Segata et al., 2011).

(4) Functional prediction via PICRUSt2: Functional abundances in the KEGG database were predicted via the PICRUSt2 tool. Differentially enriched metabolic pathways between groups were subsequently identified.

(5) Correlation analysis via heatmaps: Heatmap analysis was performed to explore the correlations between digestive enzyme activity, microbial activity, Na^+^-K^+^-ATPase activity, and Ca^2+^-Mg^2+^-ATPase activity with the intestinal microbiota in the small intestine contents.

### 3.12 Statistical methods

Statistical analyses were conducted via SPSS version 25.0. Continuous data are expressed as the means ± standard deviations. For normally distributed data, independent *t*-tests were used for pairwise comparisons, and one-way analysis of variance (ANOVA) was applied to compare means across multiple groups, followed by post-hoc pairwise comparisons using the least significant difference (LSD) test. For non-normally distributed data, the Kruskal–Wallis *H* test was used for multiple group comparisons. A significance level of *α* = 0.05 was set for all tests.

## 4 Results

### 4.1 Effects of SSP on general behavior in spleen-kidney yang deficiency IBS-D mice

**Figure 2:**
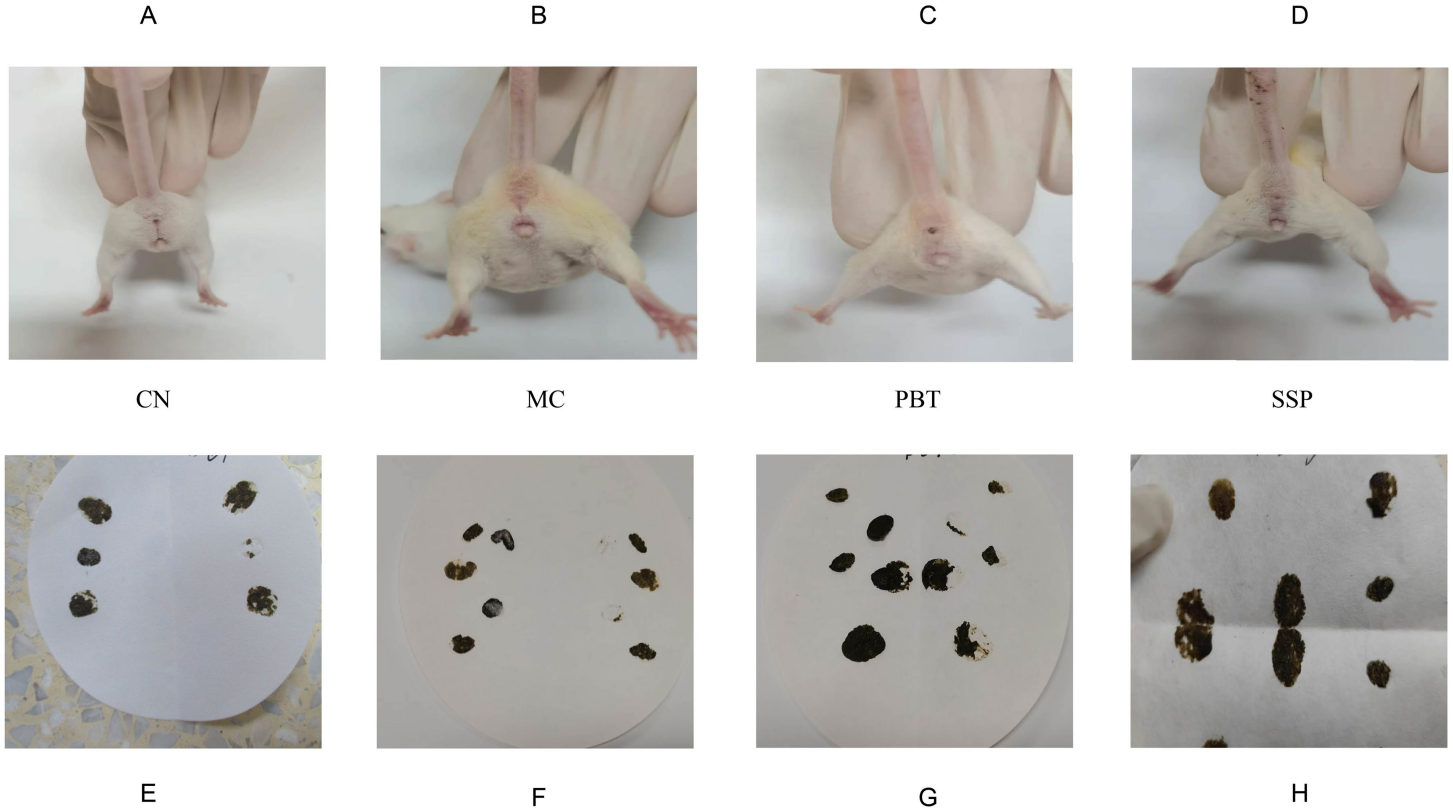
Effects of the model on fecal characteristics. **(A–D)** Perianal cleanliness, **(E–H)** Fecal characteristics. The values are expressed as the means ± standard deviations (*n* = 5 for each group). CC, normal group; MC, spontaneous recovery group; PBT, pinaverium bromide tablet group; SSP, Sishen Pill group.

The CC group of mice exhibited good mental states, were responsive, had clean perianal areas, and their feces were dark brown, moderately consistent, and maintained shape when they were picked up with tweezers without sticking. Pressing their feces on filter paper did not cause deformation or visible water stains. In contrast, model group mice were lethargic, inactive, huddled together, had arched backs, slow responses, soiled perianal fur, and loose stools. The feces deformed easily, stuck to tweezers, and caused deformation and visible water stains when pressed onto filter paper. Mice in the pinaverium bromide group showed improved mental states, increased activity, and less huddling. In SSP group, the mice became more agile with shinier fur, significantly improving their mental state and alleviating lethargy and inactivity. Their conditions resembled those of CC group, with more formed and consistent stools (Figure 2). These results suggest that SSP can improve symptoms of spleen-kidney yang deficiency in IBS-D mice.

**Figure 3:**
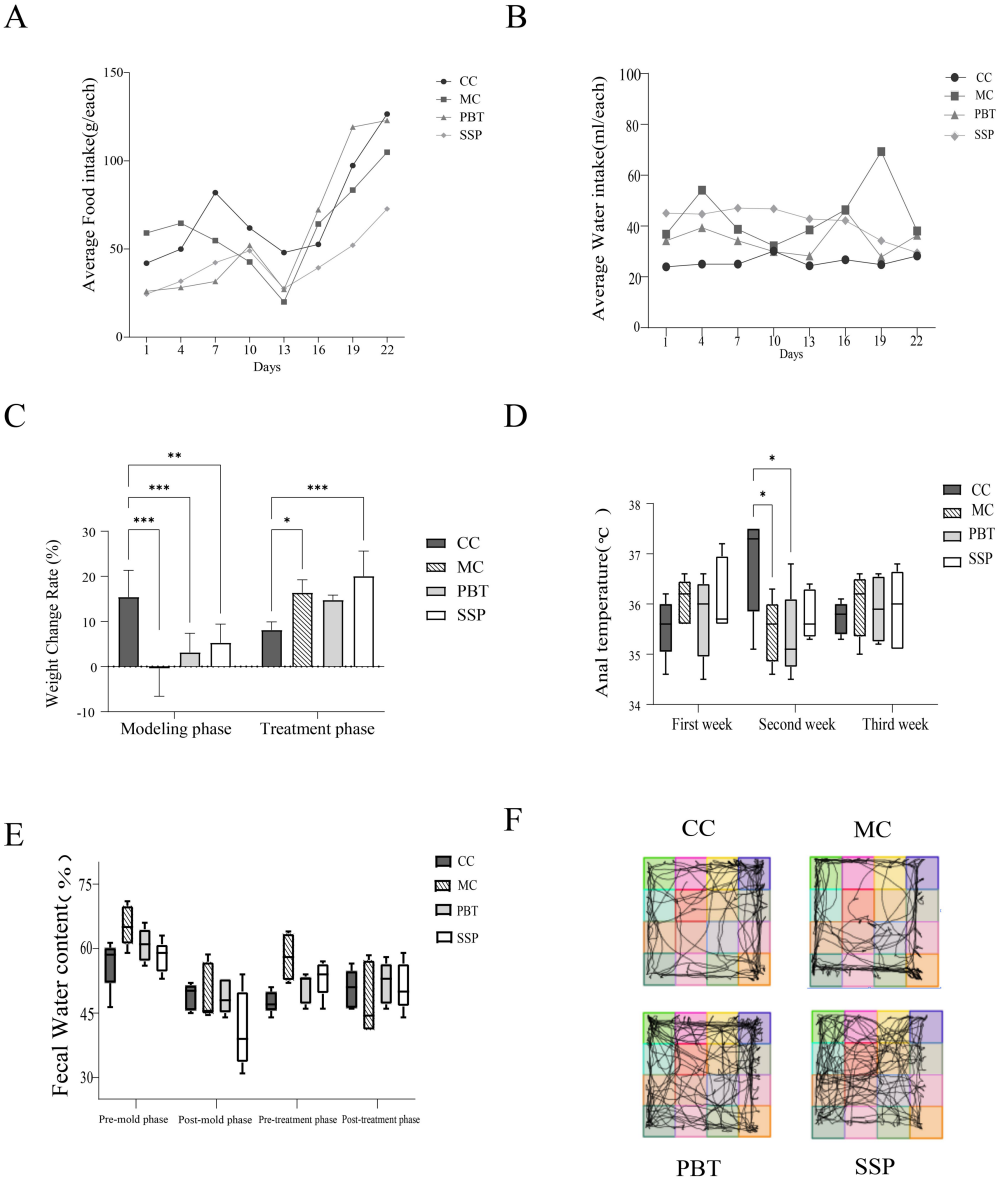
General behavioral observations and symptoms of the mice. **(A)** Average food intake, **(B)** average water intake, **(C)** weight changes, **(D)** anal temperature, **(E)** fecal water content (pre-mold: days 1–7, post-mold: days 7–14, pre-treatment: days 15–18, and post-treatment: days 19–21), **(F)** activity tracking of the mice. The values are expressed as the means ± standard deviations (*n* = 5 for each group). *p < 0.05, ** *p* < 0.01, *** *p* < 0.001 (CC, normal group; MC, spontaneous recovery group; PBT, pinaverium bromide tablet group; SSP, Sishen Pill group).

During the experiment, the average daily food intake of all four groups of mice remained stable (Figure 3A). After drug intervention, food intake significantly increased in all groups. CC group showed the smallest fluctuation in daily water intake, while MC, PBT and SSP group had higher water intake compared to CC group (Figure 3B). After SSP intervention, water intake in SSP group decreased. These results suggest that SSP has a limited effect on improving food and water intake in IBS-D mice with spleen-kidney yang deficiency. During the modeling phase, the weight change rate and anal temperature were significantly lower in all experimental groups compared to CC group (*p* < 0.05), confirming successful model establishment. In SSP group, the weight change rate and anal temperature increased, with the SSP group showing the most significant increase (Figures 3C,D). In the later stage, MC group presented a decrease in fecal water content, while both PBT and SSP groups displayed restorative changes (Figure 3E).

### 4.2 Effects of SSP on open field test results in spleen-kidney yang deficiency IBS-D mice

The mice in the CC group primarily occupied the central area of the testing box, spending less time in the corners and peripheral regions. In contrast, MC exhibited a significant decrease in grid crossings, central grid crossings, and time spent in the central grid, suggesting reduced curiosity, lethargy, decreased activity, and anxiety-depressive-like behaviors in model mice (Figure 3F). The activity patterns of PBT group and SSP group were comparable to those of CC group.

### 4.3 Effects of SSP on ATPase in spleen-kidney yang deficiency IBS-D mice

**Figure 4:**
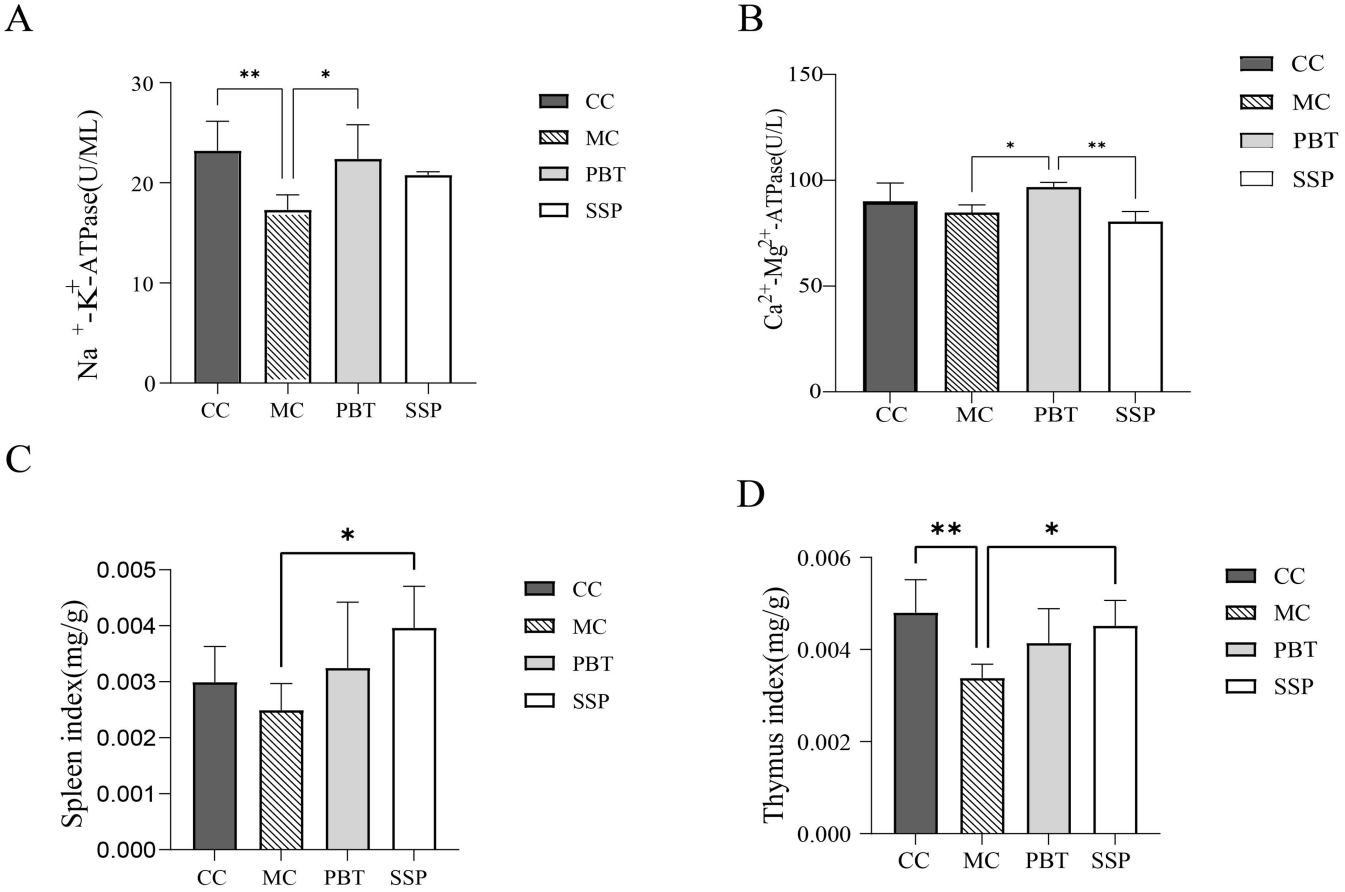
Effects of SSP on serum ELISA and organ indices in spleen-kidney yang deficiency IBS-D mice. **(A)** Na^+^-K^+^-ATPase, **(B)** Ca^2+^-Mg^2+^-ATPase, **(C)** Spleen index, and **(D)** Thymus index. The values are expressed as the means ± standard deviations (*n* = 5 for each group). *p < 0.05, **p < 0.01, ***p < 0.001 (CC, normal group; MC, spontaneous recovery group; PBT, pinaverium bromide tablet group; SSP, Sishen Pill group).

Compared to CC group, Na^+^-K^+^-ATPase and Ca^2+^-Mg^2+^-ATPase activity were slight decreased in MC group (*p* < 0.05, Figures 4A,B). After treatment, both ATPases activities significantly increased in PBT group and SSP group (*p* < 0.05). Experimental results indicated that the enhancement effect in PBT group was greater than that in SSP group. These results suggest that SSP can effectively improve the ATPase activity inhibition of ATPase activity caused by the model, but its mode of action and onset speed differ from those of pinaverium bromide.

### 4.4 Effects of SSP on organ indices in spleen-kidney yang deficiency IBS-D mice

Compared to CC group, the spleen and thymus indices were lower in MC group (*p* < 0.05). However, these indices were significantly elevated in PBT group and SSP group (Figures 4C,D), indicating that SSP decoction effectively modulates the immune response induced by the model.

### 4.5 Effects of SSP on intestinal enzyme activity in spleen-kidney yang deficiency IBS-D mice

**Figure 5:**
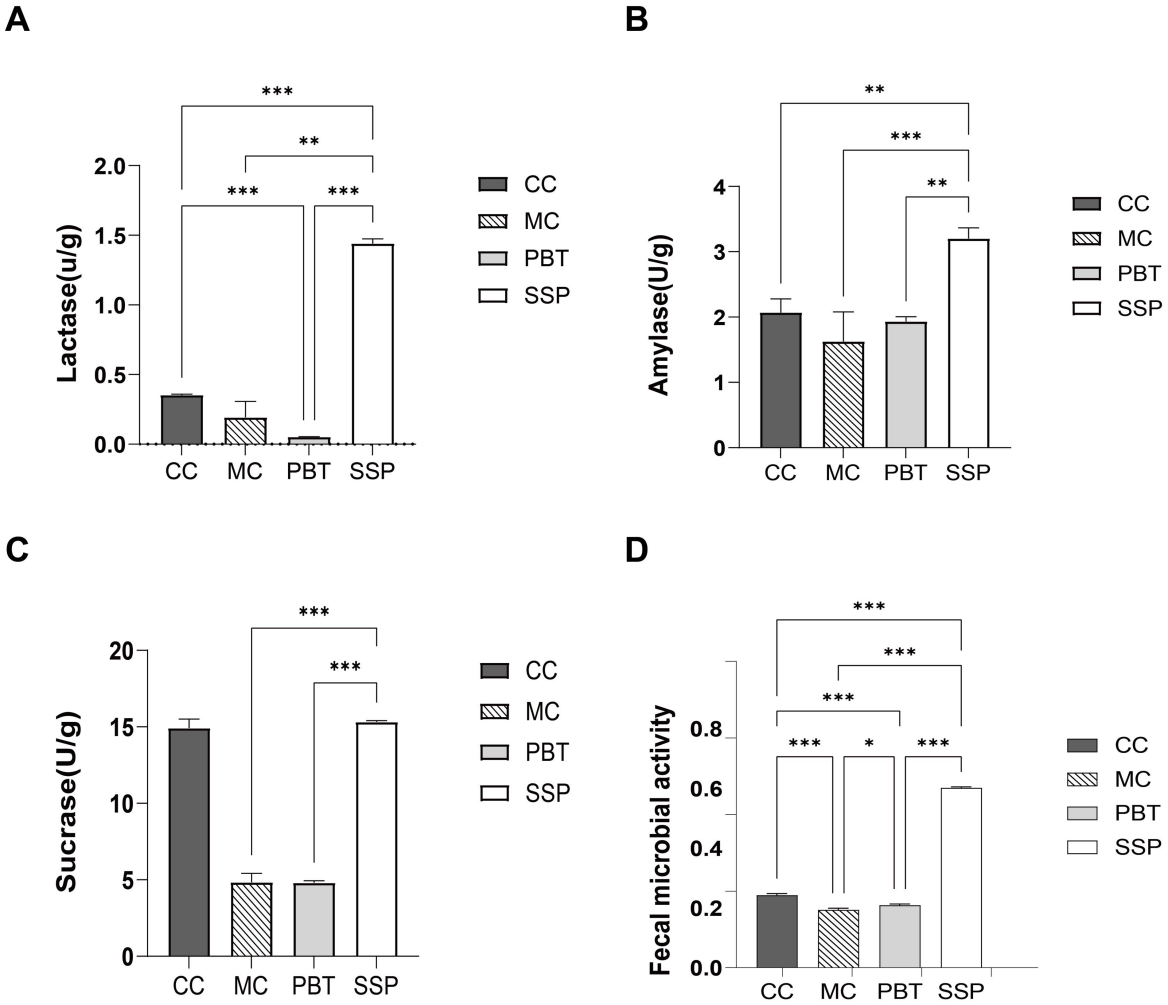
Intestinal digestive enzyme activity and microbial activity in spleen-kidney yang deficiency IBS-D mice. **(A)** Lactase. **(B)** Amylase. **(C)** Sucrase. **(D)** Fecal microbial activity. The values are expressed as the means ± standard deviations (*n* = 5 for each group). *p < 0.05, **p < 0.01, ***p < 0.001. CC, normal group; MC, spontaneous recovery group; PBT, pinaverium bromide tablet group; SSP, Sishen Pill group.

As shown in Figures 5A–C, the activities of lactase, sucrase, and amylase in MC group were lower than those in CC group (*p* < 0.05). Compared with CC group, lactase and sucrase activities were significantly lower in PBT group, while amylase activity was slightly lower. In contrast, SSP group presented significantly greater lactase and amylase activities than CC group did (*p* < 0.01), with sucrase activity increasing. These results suggest that SSP treatment significantly affects digestive enzyme activity in the intestinal contents of mice with spleen-kidney yang deficiency IBS-D.

### 4.6 Effects of SSP on intestinal microbial activity in spleen-kidney yang deficiency IBS-D mice

Compared to CC group, microbial activity was significantly lower in MC group (*p* < 0.01). In PBT group, microbial activity decreased (*p* < 0.01), while it significantly increased in SSP group (*p* < 0.01) (Figure 5D).

### 4.7 Changes of SSP on the microbial community of the small intestinal contents of spleen-kidney yang deficiency IBS-D mice

#### 4.7.1 Effects of SSP on ASV count and rarefaction curves in spleen-kidney yang deficiency IBS-D mice

**Figure 6:**
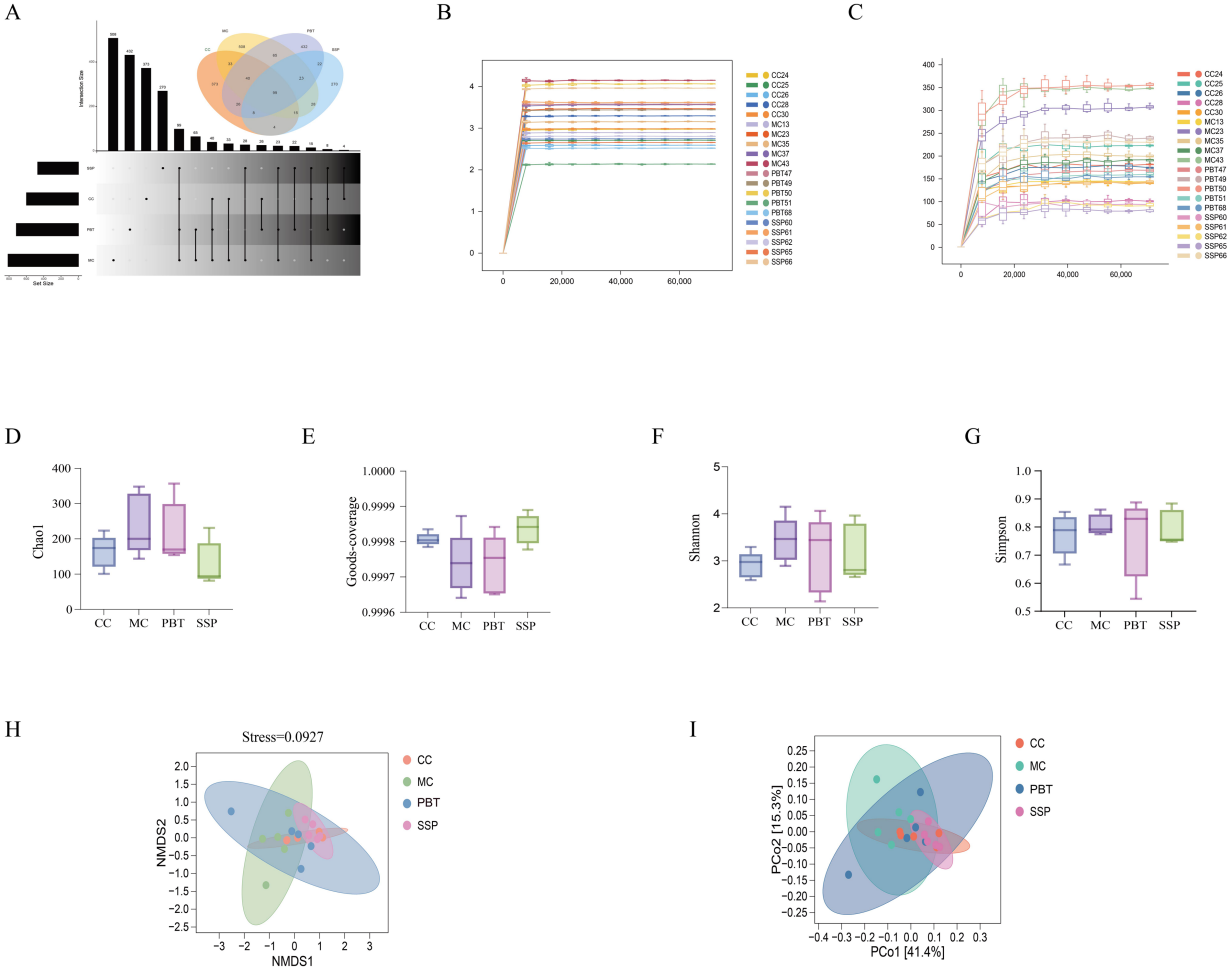
Analysis of the microbiota structure of the intestinal microbiota. **(A)** Advanced Venn diagram, **(B)** rarefaction curve, **(C)** Shannon–Wiener curve, **(D)** Chao1 index, **(E)** Good’s coverage index, **(F)** Shannon index, **(G)** Simpson index, **(H)** NMDS analysis, **(I)** PCoA (CC, normal group; MC, spontaneous recovery group; PBT, pinaverium bromide tablet group; SSP, Sishen Pill group).

The Venn diagram analysis (Figure 6A) revealed that CC group contained 472 ASVs, with 373 unique ASVs; MC group included 607 ASVs, with 508 unique ASVs; PBT group included 531 ASVs, with 432 unique ASVs; and SSP group included 369 ASVs, with 270 unique ASVs. The rarefaction curve, which was used to assess sequencing depth and species richness, plateaued as sequencing depth increased, indicating that the depth was sufficient to capture nearly all species in the samples from all four groups (Figures 6B,C).

#### 4.7.2 Changes in α diversity of intestinal microbiota in spleen-kidney yang deficiency IBS-D mice

The α diversity analysis of the abundance rank curve indicated that MC and PBT groups presented longer horizontal axis lengths than did the other groups did, suggesting a greater number of ASVs in these groups. Additionally, the curves for MC group and PBT group were flatter than those for CC group and SSP group were, indicating smaller variations in ASV abundance and a more uniform composition. As shown in Figures 6D–G, the diversity indices of intestinal content samples varied among the groups. Compared to CC group, MC group showed increases in Chao1, Shannon, and Simpson indices; SSP group exhibited an increase in Simpson index; and PBT group demonstrated increases in Chao1 and Simpson indices. However, none of these differences reached statistical significance (*p* > 0.05).

#### 4.7.3 Changes in β diversity of the intestinal microbiota in spleen-kidney yang deficiency IBS-D mice

The first principal component explained 41.4% of the variance, and the second principal component explained 15.3% (Figure 6I). Compared with the broad distributions of MC group and PBT group, those of CC group and SSP group were more concentrated, indicating that model induction affects the structure of the intestinal microbiota. The stress value obtained from NMDS analysis was 0.0927 (Figure 6H).

#### 4.7.4 Effects of the relative abundance of intestinal contents in spleen-kidney yang deficiency IBS-D mouse

**Figure 7:**
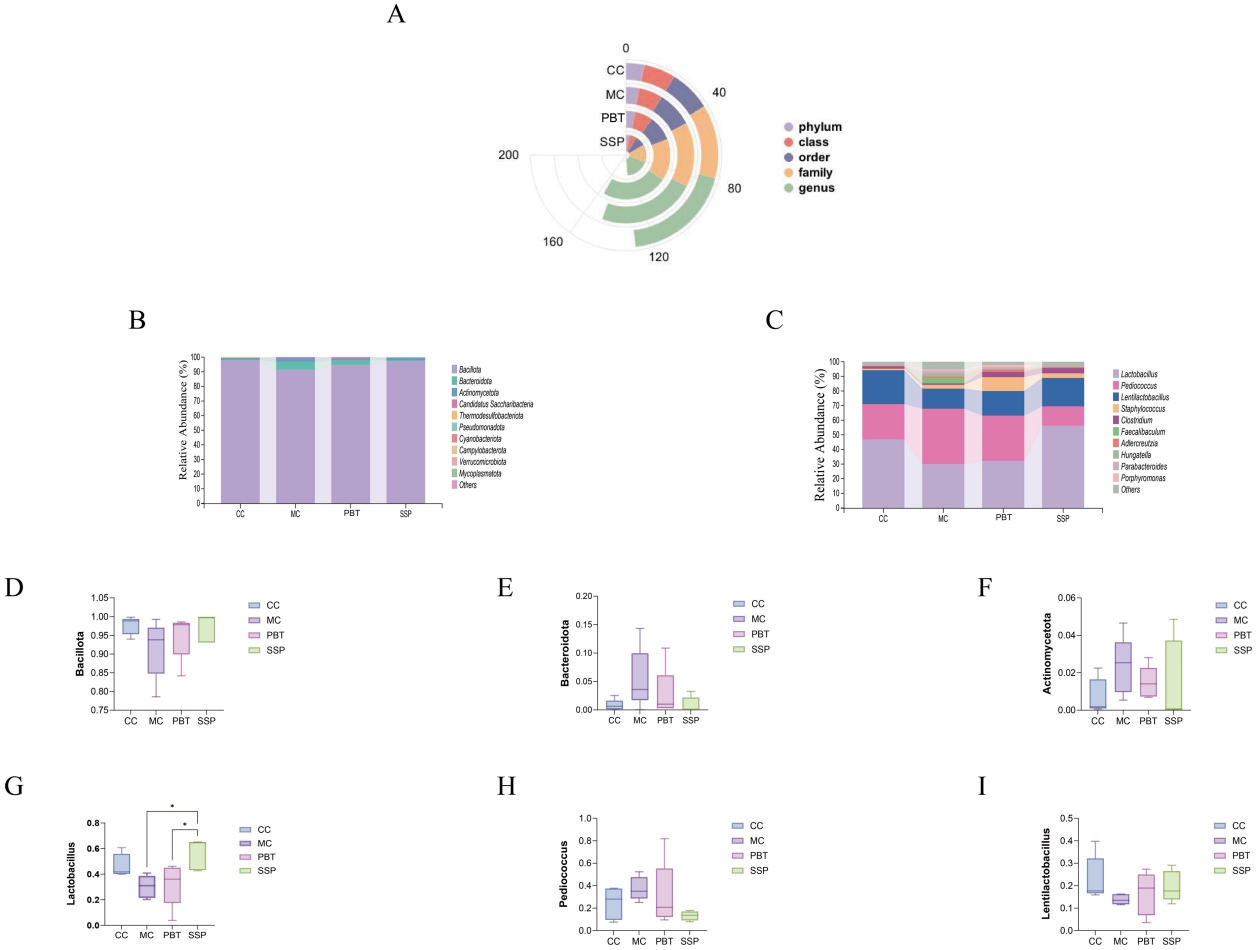
The composition of the intestinal microbiota in mice. **(A)** Radial bar chart. **(B)** Phylum-level relative abundance plot. **(C)** Genus-level relative abundance plot. **(D–F)** Phylum-level dominant intestinal microbiota. **(G–I)** Genus-level dominant intestinal microbiota. The values are expressed as the means ± standard deviations (*n* = 5 for each group). *p < 0.05. CC, normal group; MC, spontaneous recovery group; PBT, pinaverium bromide tablet group; SSP, Sishen Pill group.

A comparison of taxonomic changes after treatment revealed that SSP group presented lower microbial abundance across all taxonomic categories (Figure 7A). On the basis of the intestinal microbiota data, we selected the top 10 most abundant phyla and genera for further analysis, focusing on their frequency and relative abundance. At the phylum level, the dominant phyla included Bacillota, Bacteroidota, Actinomycetota, Candidatus sanormol group (a subgroup of haribacteria), and Thermodesulfobacteriota. Compared to MC group, the relative abundances of Bacillota and Actinomycetota were significantly greater in the other three treatment groups (*p* < 0.05), while Bacteroidota abundance was significantly lower (*p* < 0.05). The relative abundance of Candidatus sanormol group was lower in both normal and SSP groups compared to MC group but higher in PBT group. The abundance of Thermodesulfobacteriota was notably greater in CC group, though the difference was not statistically significant (Figure 7B).

At the genus level, the dominant genera included *Lactobacillus*, *Pediococcus*, *Staphylococcus*, and *Clostridium*. Compared to MC group, the relative abundances of *Lactobacillus*, *Lactobacillus*, and *Clostridium* were increased in the other three groups, while *Pediococcus* abundance was lower (Figure 7C). Notably, the abundance of *Staphylococcus* was greater in both SSP group and PBT group than in MC group, while it slightly decreased in CC group.

#### 4.7.5 Analysis of dominant intestinal microbiota in spleen-kidney yang deficiency IBS-D mice

Compared to MC group, SSP group presented a 5.65% increase in the relative abundance of Bacillota, a 4.5% decrease in Bacteroidota, and a 0.84% decrease in Actinomycetota (Figures 7D–F). At the genus level, the relative abundance of *Lactobacillus* in SSP group increased by 25.93%, while that of *Lentilactobacillus* increased by 5.82%, and *Pediococcus* decreased by 24.29% (Figures 7G–I). These results indicate that SSP intervention significantly altered the composition of dominant intestinal microbiota at both the phylum and genus levels.

#### 4.7.6 Analysis of characteristic intestinal microbiota in spleen-kidney yang deficiency IBS-D mice

**Figure 8:**
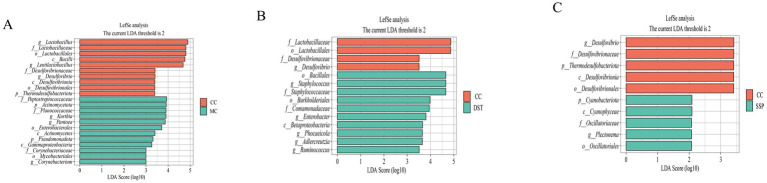
Analysis of characteristic bacteria in the small intestinal contents of IBS-D Mice treated with SSP for spleen-kidney yang deficiency intervention. **(A)** Distribution of LDA scores (CC vs. MC); **(B)** distribution of LDA scores (CC vs. PBT); **(C)** distribution of LDA scores (CC vs. SSP); (CC, normal group; MC, spontaneous recovery group; PBT, pinaverium bromide tablet group; SSP, Sishen Pill group).

We conducted LEfSe analysis (LDA score > 2) to identify key microbial taxa, as shown in the hierarchical taxonomy of the figure. In the comparison between CC group and MC group (Figure 8A), *Lactobacillus, Lentilactobacillus*, and *Desulfovibrio* were characteristic of CC group, while *Corynebacterium*, *Kurthia*, and *Pantoea* were specific to MC. In the comparison between CC group and PBT group (Figure 8B), *Desulfovibrio* was characteristic of CC group, whereas *Staphyloconormol groupus*, *Enterobacter*, *Phocaeicola*, *Adlercreutzia*, and *Ruminoconormol groupus* were characteristic of PBT group. In the comparison between CC group and SSP group (Figure 8C), *Desulfovibrio* was characteristic of CC group, and *Plectonema* was specific to SSP group.

**Figure 9:**
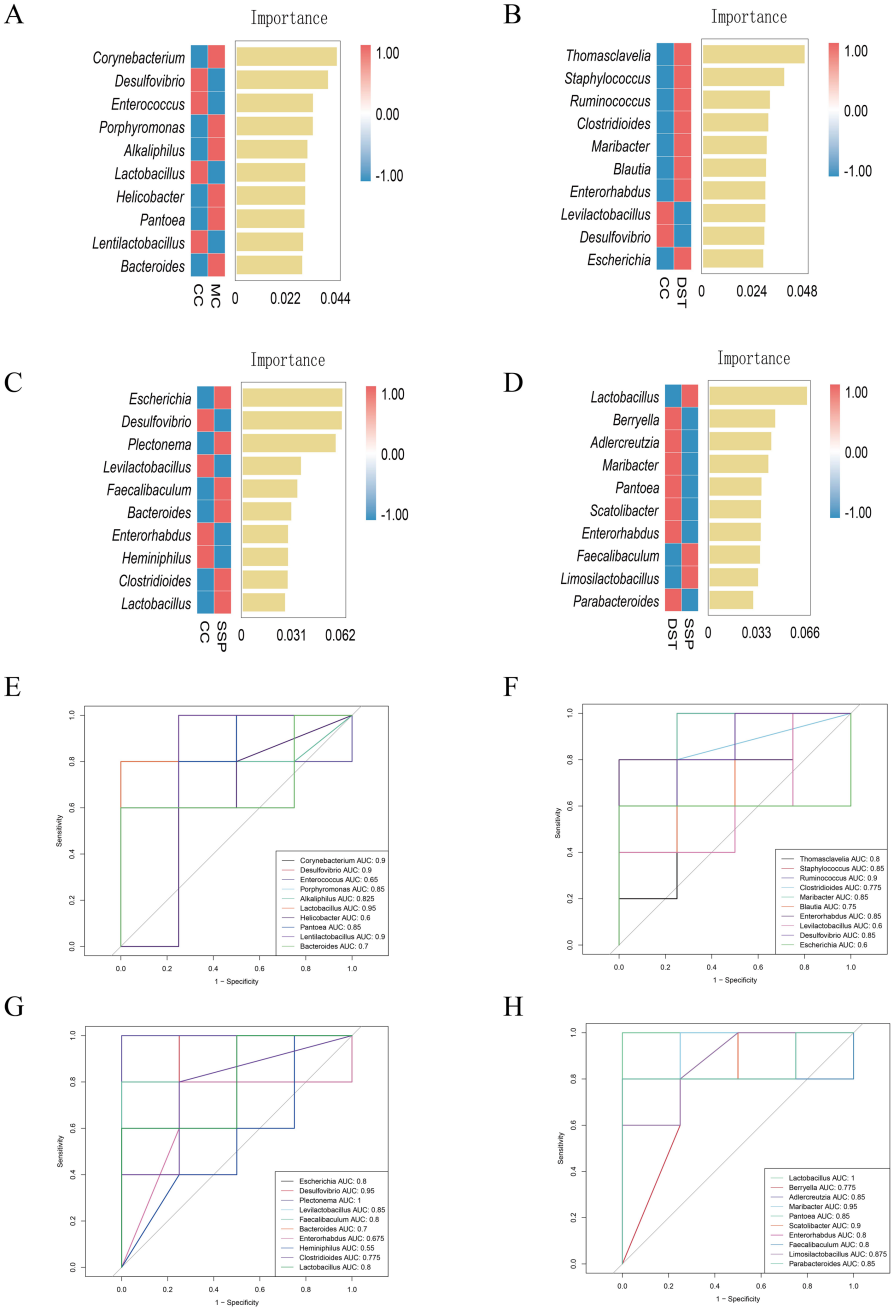
Analysis of characteristic bacteria in the small intestinal contents of IBS-D Mice treated with SSP for spleen-kidney yang deficiency intervention. **(A)** Genus-level random forest plot (CC vs. MC); **(B)** genus-level random forest plot (CC vs. PBT); **(C)** genus-level random forest plot (CC vs. SSP); **(D)** genus-level random forest plot (PBT vs. SSP); **(E)** genus-level ROC curve (CC vs. MC); **(F)** genus-level ROC curve (CC vs. PBT); **(G)** genus-level ROC curve (CC vs. SSP); **(H)** genus-level ROC curve (PBT vs. SSP) (CC, normal group; MC, spontaneous recovery group; PBT, pinaverium bromide tablet group; SSP, Sishen Pill group).

To further identify key species differentiating the four groups, we constructed a random forest model to explore the non-linear relationships between variables (Figures 9A–D). ROC curve analysis of the results from the random forest model, with an AUC > 0.8, validated the diagnostic accuracy and the effectiveness of using characteristic bacteria for group classification.

In the ROC analysis comparing CC group and MC group (Figure 9E), the abundances of *Desulfovibrio* (AUC = 0.9), *Lactobacillus* (AUC = 0.95), and *Lentilactobacillus* (AUC = 0.9) in CC group were all greater than 0.8. Similarly, *Corynebacterium* (AUC = 0.9), *Porphyromonas* (AUC = 0.85), *Alkaliphilus* (AUC = 0.825), and *Pantoea* (AUC = 0.85) in MC also had AUC values above 0.8. In the ROC analysis comparing CC and PBT (Figure 9F), *Desulfovibrio* (AUC = 0.85) in CC group had an AUC greater than 0.8, while in PBT group, *Staphyloconormol groupus* (AUC = 0.85), *Ruminoconormol groupus* (AUC = 0.9), *Maribacter* (AUC = 0.85), and *Enterorhabdus* (AUC = 0.85) all had greater than 0.8. In the ROC analysis between CC group and SSP group (Figure 9G), the abundances of *Desulfovibrio* (AUC = 0.95) and *Levilactobacillus* (AUC = 0.85) in CC group both exceeded 0.8. In SSP group, *Plectonema* (AUC = 1) was above 0.8. In the ROC analysis between PBT and SSP groups (Figure 9H), *Adlercreutzia* (AUC = 0.85), *Maribacter* (AUC = 0.95), *Pantoea* (AUC = 0.85), *Scatolibacter* (AUC = 0.9), and *Parabacteroides* (AUC = 0.85) in PBT group had AUC values above 0.8. In SSP group, *Lactobacillus* (AUC = 1.0) and *Limosilactobacillus* (AUC = 0.875) also exceeded 0.8. These findings indicate that the selected bacterial genera exhibit strong diagnostic sensitivity. Specifically, *Desulfovibrio* served as diagnostic biomarkers for CC group; *Corynebacterium, Porphyromonas, Alkaliphilus* and *Pantoea* for MC group; *Staphyloconormol groupus*, *Ruminoconormol groupus*, *Maribacter*, *Adlercreutzia, Pantoea, Scatolibacter,* and *Enterorhabdus* served as diagnostic biomarkers for PBT group; *Plectonema* and *Lactobacillus* served as diagnostic biomarkers for SSP group.

Through integrated analyses using LEfSe, random forest, and ROC curves, the characteristic microbiota of each group were summarized as follows: (1) *Desulfovibrio* for CC group; (2) *Corynebacterium* and *Pantoea* for MC group; (3) *Staphyloconormol groupus, Enterobacter, Phocaeicola, Adlercreutzia, Ruminoconormol groupus* for PBT group; and (4) *Plectonema* and *Lactobacillus* for SSP group.

#### 4.7.7 Functional analysis of the intestinal microbiota in spleen-kidney yang deficiency IBS-D mice

**Figure 10:**
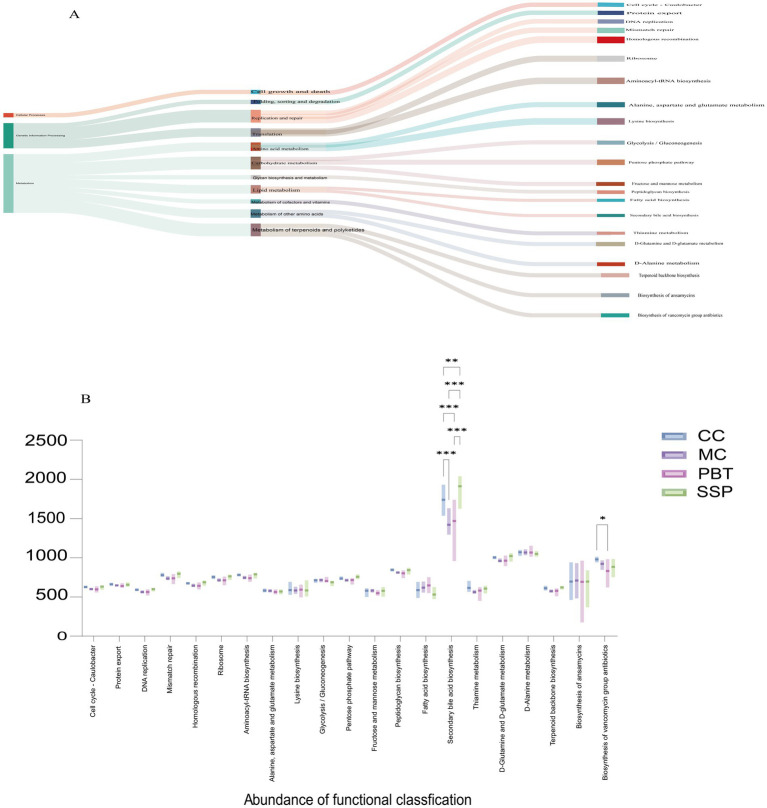
Characteristics of the intestinal microbiota. **(A)** Evolutionary branching diagrams, **(B)** metabolic function intergroup comparative box line plots (Level 3). (*n* = 5 for each group, *p < 0.05, **p < 0.01, ***p < 0.001). CC, normal group; MC, spontaneous recovery group; PBT, pinaverium bromide tablet group; SSP, Sishen Pill group.

Analysis of the metabolic and functional changes in the intestinal microbiota revealed three main functional categories, with metabolism being the most prominent. Among the top 21 KEGG pathways analyzed, 76.19% were metabolism related (Figure 10A). These pathways primarily affect nucleotide, energy, amino acid, and carbohydrate metabolism, with secondary bile acid metabolism resulting in particularly high activity (Figure 10B). Compared to CC group, MC group presented a significant decrease in secondary bile acid metabolism (*p* < 0.001). After SSP treatment, secondary bile acid metabolism was restored and even surpassed that in CC group levels (*p* < 0.01).

#### 4.7.8 Correlation analysis of the intestinal microbiota

**Figure 11:**
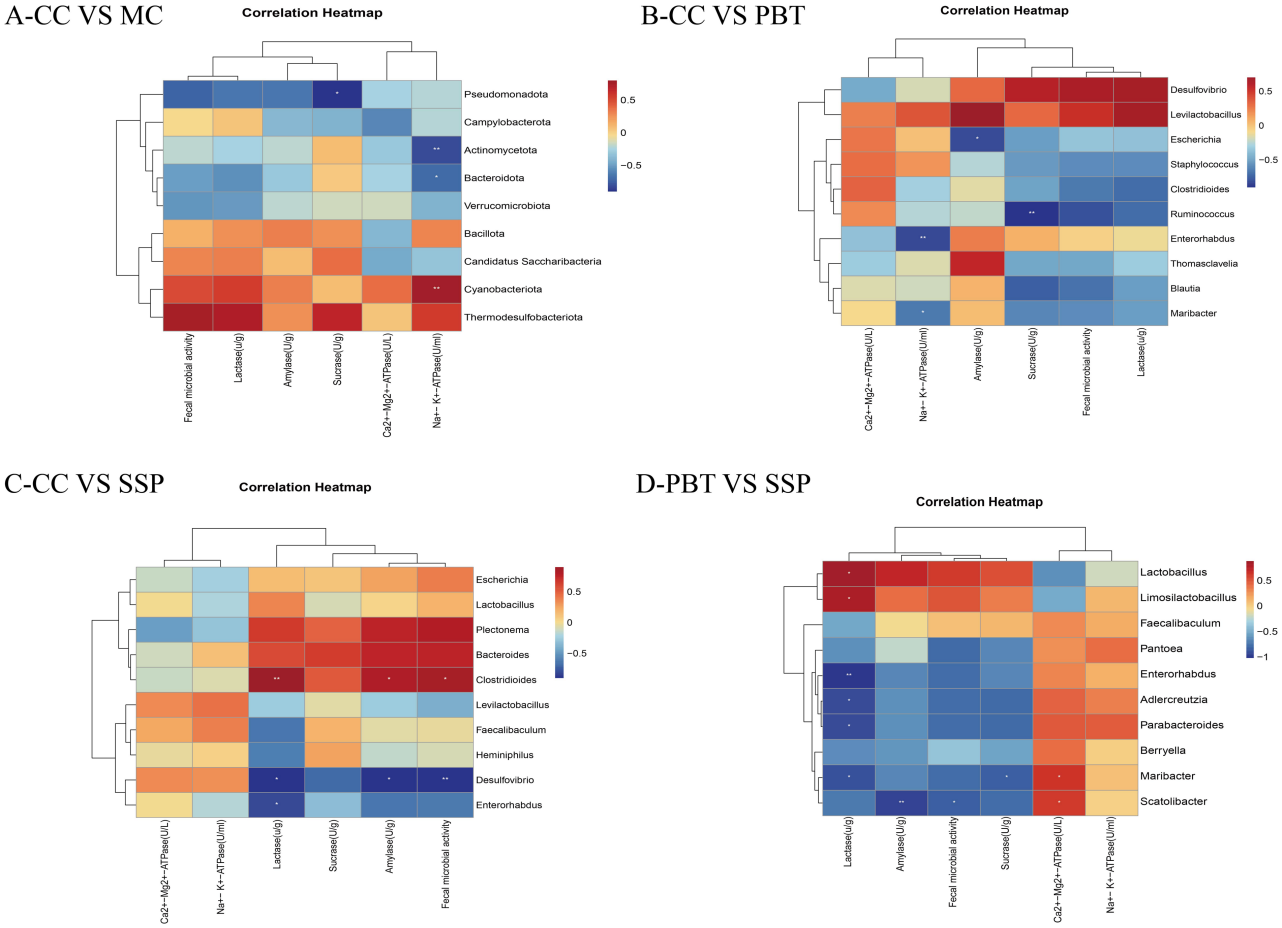
Correlation analysis of characteristic bacteria in small intestinal contents of IBS-D mice with spleen-kidney yang deficiency intervened by SSP and the activities of Na^+^-K^+^-ATPase, Ca^2+^-Mg^2+^-ATPase, and intestinal digestive enzymes. **(A)** Correlation heatmap between CC and MC; **(B)** correlation heatmap between CC and PBT; **(C)** correlation heatmap between CC and SSP; **(D)** Correlation heatmap between PBT and SSP (CC, normal group; MC, spontaneous recovery group; PBT, pinaverium bromide tablet group; SSP, Sishen Pill group. *p < 0.05, **p < 0.01).

To explore the correlation between the characteristic microbiota and various indicators, a correlation analysis was conducted for each group. In the comparison between CC group and MC group (Figure 11A), *Pseudomonadota* was negatively correlated with sucrase (*p* < 0.05), *Actinomycetota* was significantly negatively correlated with Na^+^-K^+^-ATPase (*p* < 0.01), Bacteroidota was negatively correlated with Na^+^-K^+^-ATPase (*p* < 0.05), and *Cyanobacteriota* was significantly positively correlated with Na^+^-K^+^-ATP-ase (*p* < 0.01). In the comparison between CC group and PBT group (Figure 11B), *Enterorhabdus* was significantly negatively correlated with Na^+^-K^+^-ATPase (*p* < 0.01), *Maribacter* was negatively correlated with Na^+^-K^+^-ATPase (*p* < 0.05), *Escherichia* was negatively correlated with amylase (*p* < 0.05), and *Ruminoconormol groupus* was significantly negatively correlated with sucrase (*p* < 0.01). In the comparison between CC group and SSP group (Figure 11C), *Clostridioides* was significantly positively correlated with lactase (*p* < 0.01) and positively correlated with amylase and intestinal microbial activity (*p* < 0.05), while *Desulfovibrio* was significantly negatively correlated with intestinal microbial activity (*p* < 0.01) and negatively correlated with lactase and amylase, and *Enterorhabdus* was negatively correlated with lactase (*p* < 0.05). In the comparison between PBT group and SSP group (Figure 11D), Ca^2+^-Mg^2+^-ATPase was positively correlated with *Maribacter* and *Scatolibacter* (*p* < 0.05). Lactase was positively correlated with *Lactobacillus* and *Limosilactobacillus* (*p* < 0.05), but negatively correlated with *Enterorhabdus* (*p* < 0.05). Additionally, Lactase was negatively correlated with *Adlercreutzia*, *Parabacteroides*, and *Maribacter* (*p* < 0.05). Amylase activity was significantly negatively correlated with the abundance of *Scatolibacter* (*p* < 0.01). Fecal microbial activity was negatively correlated with *the abundance of Scatolibacter* (*p* < 0.01), and sucrase activity was negatively correlated with the abundance of *Maribacter* (*p* < 0.05).

## 5 Discussion

### 5.1 Therapeutic effects of SSP on general behavioral characteristics in spleen-kidney yang deficiency IBS-D mice

IBS-D can be categorized into four basic types: liver depression and spleen deficiency syndrome, spleen deficiency with dampness syndrome, spleen-kidney yang deficiency syndrome, and damp-heat syndrome in the spleen and stomach, and cold-heat complex syndrome (Fu et al., 2023). Among these, spleen-kidney yang deficiency syndrome is common, with symptoms such as watery stools, undigested food, abdominal pain relieved by warmth, lumbar soreness, and cold extremities. The primary treatment for this syndrome is the SSP formula. In SSP, *P. corylifolia* warms and supplements kidney yang, while *E. rutaecarpa* expels cold and restores the functions of both the kidney and spleen. *M. fragrans* warms the stomach and astringes the intestines, while *Schisandra chinensis* helps stop diarrhea by astringing the essence. *Z. officinale* disperses water Qi, and *Z. jujuba* nourishes the spleen, dispels dampness, and protects the stomach. Together, these herbs work synergistically to warm and supplement the spleen and kidney, astringe the intestines, and alleviate diarrhea. Modern pharmacological studies (Chen et al., 2015; Tao et al., 2019) have shown that SSP protects intestinal function, possesses anti-diarrheal and anti-inflammatory effects, regulates the microbiota, inhibits spontaneous intestinal activity, and improves energy metabolism. Network pharmacology studies have also indicated that SSP possesses 116 potential targets and 5 core targets for treating IBS-D. The core bioactive compounds include bergapten and β-sitosterol, which are involved primarily in signaling pathways such as pathways in cancer, IL-17 and TNF (Shen and Guan, 2022).

In this study, we successfully replicated the spleen-kidney yang deficiency IBS-D model via adenine and *Folium senna* combined with restraint-tail clamping stimulation. The model group displayed characteristic symptoms, such as huddling, lethargy, increased fecal water content, and reduced anal temperature, confirming successful establishment of the model. Compared with those in CC group, rectal temperature and fecal water content of the mice in SSP group significantly increased, indicating improved diarrhea and yang deficiency symptoms. Additionally, symptoms such as cold aversion, preference for huddling, and hunched posture were alleviated. The gradual normalization of diet, water intake, rectal temperature, and body weight was observed. These results suggest that SSP decoction can partially improve the general behavior and physiological functions of mice with spleen-kidney yang deficiency IBS-D.

### 5.2 Therapeutic effects of SSP on energy embolism in spleen-kidney yang deficiency IBS-D mice

Na^+^-K^+^-ATPase and Ca^2+^-Mg^2+^-ATPase are essential enzymes for maintaining cellular energy metabolism. They play a critical role in regulating cell osmotic pressure, membrane potential, ion transport, and nutrient absorption, all of which are vital for the proper functioning of intestinal epithelial cells (Zhu, 2014). When energy metabolism is impaired, the activities of Na^+^-K^+^-ATPase and Ca^2+^-Mg^2+^-ATPase are suppressed, leading to intracellular overload of Na^+^ and Ca^2+^. This overload further exacerbates the energy deficit and induces cellular damage (Pongkorpsakol et al., 2017). In the intestinal context, this pathological change inhibits Na^+^-K^+^-ATPase-dependent transepithelial ion transport (Magalhães et al., 2016), thereby impairing water absorption from the intestinal lumen and ultimately contributing to diarrhea. In kidney-yang deficiency diarrhea models, decreased ATPase activity suggests that mitochondrial dysfunction affects energy metabolism (Zhu et al., 2022). In this study, the activities of Na^+^-K^+^-ATPase and Ca^2+^-Mg^2+^-ATPase were decreased in the IBS-D model with spleen-kidney yang deficiency, which is consistent with previous findings. Treatment with SSP elevated the serum activities of these ATPases, improved energy metabolism levels, and alleviated diarrheal symptoms (Zhu et al., 2023). SSP likely alleviates diarrhea in kidney-yang deficiency by increasing the activities of Na^+^-K^+^-ATPase and Ca^2+^-Mg^2+^-ATPase, promoting the expression of colonic aquaporins (AQP3, AQP4), and enhancing intestinal water absorption (Lv, 2020). Our experimental results revealed that both pinaverium bromide and SSP increased serum ATPase activity, with a more pronounced effect observed in the pinaverium bromide group. This difference may be due to the rapid onset of pinaverium bromide, which directly targets ion channels, while the holistic regulatory effects of SSP require a longer time to manifest.

The intestinal microbiota directly participates in host digestion by secreting a variety of digestive enzymes, including amylases, sucrases, and proteases. These enzymes play pivotal roles in the digestion and absorption of nutrients, and their activity levels serve as crucial indicators for assessing intestinal functional status (He et al., 2019). Furthermore, they help preserve the integrity of the gut epithelial barrier and promote water and electrolyte absorption (Guo et al., 2019). Microbial metabolites, particularly short-chain fatty acids (SCFAs), are integral to gut energy metabolism. Butyrate, a key SCFA, serves as the primary energy source for colonic epithelial cells and is essential for maintaining barrier integrity, suppressing pathogens, and promoting probiotic colonization (Lin et al., 2023). Dysbiosis, characterized by a decline in butyrate producers such as *Clostridium butyricum*, reduces SCFA production (Hu et al., 2020), leading to impaired ATP synthesis and subsequent suppression of ATPase activity in the colonic epithelium. Previous work from our team has shown that an animal model of diarrhea with kidney-yang deficiency is characterized by dysfunction in multiple digestive enzymes (Zhou et al., 2024). This dysfunction was significantly rescued by SSP treatment (Zhou et al., 2025), demonstrating its therapeutic potential in restoring digestive function.

In this study, we further measured the activities of lactase, sucrase, and amylase. The results indicated that the activities of all three enzymes were significantly reduced after modeling, confirming that the model successfully replicated a state of impaired intestinal digestive function. After SSP treatment, the activities of these enzymes significantly increased, with some even returning to levels equal to or greater than those of the normal group. These findings demonstrate that SSP effectively reverses the digestive enzyme dysfunction associated with spleen-kidney yang deficiency IBS-D. In conclusion, SSP can restore energy metabolism in spleen-kidney yang deficiency IBS-D by regulating both ATPase and digestive enzyme activities.

### 5.3 Therapeutic effects of SSP on intestinal microbiota in spleen-kidney yang deficiency IBS-D mice

The intestinal microbiota, a crucial component of intestinal microecology, plays a key role in maintaining intestinal health, regulating immunity, promoting gastrointestinal function, and producing metabolic products due to its diverse microbial community. Dysbiosis of the microbiota is strongly associated with an increased risk of IBS (Zhang et al., 2024). Our previous studies demonstrated that combined restraint and tail-clamping stimulation accelerates gastrointestinal motility and induces inflammation, leading to IBS via increased visceral hypersensitivity (Deng et al., 2025). Bioinformatic analysis of small intestinal contents revealed compositional changes in the intestinal microbiota of IBS-D mice with spleen-kidney yang deficiency. The model group presented increased microbial abundance, which was modulated by both SSP and pinaverium bromide treatments, with SSP generally resulting in lower abundance. While *α* diversity was not significantly different, significant β diversity shifts were observed. PCoA analysis revealed that SSP treatment led to a tighter clustering of samples, indicating a more consistent microbial community structure post-treatment, suggesting a targeted modification of specific taxa rather than overall diversity. In a study by Liu et al. (2019), treatment with SSP in IBS-D mice led to a reduction in Proteobacteria and *Mycoplasma*, and an increase in *Clostridia*, *Turicibacter*, and *Romboutsia*, which effectively alleviated IBS-D symptoms. Furthermore, an integrated analysis incorporating LEfSe, random forest, and ROC curves identified *Plectonema* and *Lactobacillus* as characteristic genera in SSP group. While *Plectonema* is frequently studied in agricultural contexts, it has been reported to play significant roles in biochemical regulation (Shahid et al., 2022). *Lactobacillus*, a well-known beneficial bacterium, produces lactic acid in the gut and is renowned for its health benefits, particularly in alleviating diarrhea (Parker et al., 2020). Some reports indicate that *Lactobacillus* act as psychobiotics, exerting protective effects against intestinal inflammation and playing an important role in maintaining mental health (Xie et al., 2022). Collectively, these findings suggest that SSP ameliorates IBS-D by modulating intestinal microbiota balance—specifically by increasing the relative abundance of certain probiotics and decreasing that of harmful bacteria—thereby restoring a microbial composition closer to that of CC group.

Our study revealed a marked decrease in secondary bile acid (BA) biosynthesis pathway activity (*p* < 0.001) in IBS-D model with spleen-kidney yang deficiency. This observation is consistent with prior findings of gut microbiota dysbiosis and bile acid malabsorption in this model (Han, 2019). Bile acids, as metabolites of the gut microbiota, interact with microbes in a way that is crucial to IBS-D pathogenesis. The transformation of primary to secondary BAs relies heavily on bacterial processes like deconjugation and 7α-dehydroxylation (Long et al., 2017). Therefore, dysbiosis likely reduces key functional bacteria, leading to impaired secondary BA metabolism. This disruption is pathologically significant, as abnormal secondary BA metabolism can impair the intestinal barrier and enhance colonic secretion, ultimately resulting in diarrhea (Lacy et al., 2021). Treatment with SSP significantly reversed the impaired secondary BA metabolism. Notably, the pinaverium bromide group presented even lower secondary BA levels than the normal control. We hypothesize that SSP restores the normal BA metabolic cycle by reconstructing the intestinal microbiota. This regulation is also likely to contribute to the restoration of intestinal barrier integrity and a reduction in BA-driven mucosal activation (Fogelson et al., 2023), thus alleviating diarrhea. In conclusion, regulation of the “intestinal microbiota-bile acid metabolism” axis is a key mechanism underlying SSP’s efficacy.

LEfSe, random forest, and ROC curve analyses identified characteristic microbiota for each group: *Lactobacillus* and *Desulfovibrio* were prominent in CC group, while *Corynebacterium* and *Pantoea* were characteristic of MC group. *Plectonema* was a key feature of SSP group. *Lentilactobacillus*, a species of *Lactobacillus*, regulates inflammatory cytokines, decreases the levels of proinflammatory mediators and promotes anti-inflammatory responses, effectively alleviating IBS symptoms. *Corynebacterium* and *Pantoea* were characteristic microbiota in MC group. *Corynebacterium* frequently appears in invasive infections and is part of the endogenous skin microbiota (Streifel et al., 2022). *Pantoea* is a multifunctional bacterial genus with plant and animal pathogenic strains, and research on its effects on human intestinal function is currently insufficient and requires further investigation (Shetty et al., 2024). These findings indicate that SSP can restore the intestinal microbiota to normal levels by adjusting the changes in the intestinal microbiota.

### 5.4 Correlation between intestinal microbiota changes and ATPase activity in the treatment of spleen-kidney yang deficiency IBS-D with SSP

To investigate the microecological mechanism by which SSP influences ATPase activity via intestinal microbiota modulation, we constructed correlation networks between gut microbiota and ATPase activity across different groups. The results revealed that the microbiota-ATPase correlation profile formed after SSP intervention was distinctly different from those of MC group and PBT group, suggesting that SSP restores intestinal energy metabolism through a unique pathway.

In the correlation analysis between CC group and SSP group, significant correlations were observed between microbial shifts and both digestive enzyme activity and microbial metabolic activity, however, their direct associations with ATPase activity did not reach statistical significance. *Clostridioides* was significantly positively correlated with lactase activity (*p* < 0.01), whereas *Desulfovibrio* was significantly negatively correlated with fecal microbial activity (*p* < 0.01). *Clostridioides* comprises both toxin-producing and non-toxin-producing strains. It has been reported that an increase in butyrate-producing bacteria, such as *Lachnoclostridium* and *Clostridioides*, can exert neuroprotective effects by promoting butyrate synthesis (Duan et al., 2023), which aligns with our experimental observations. *Desulfovibrio*, a gram-negative anaerobic bacterium, is the most common sulfate-reducing bacterium in the human body. It is considered an opportunistic pathogen that may overgrow in the context of various intestinal and extra-intestinal diseases (Singh et al., 2023). *Desulfovibrio* can produce lipopolysaccharide in the bloodstream, activating Toll-like receptor 4 (TLR4)-dependent signaling pathways and exacerbating systemic inflammation (Petersen et al., 2019). Given that intestinal microbial activity broadly reflects the overall metabolic capacity of the gut microbiota through a positive feedback mechanism, it is plausible to speculate on an intrinsic link between *Desulfovibrio* and fecal microbial activity, although this area requires further investigation. This indirect regulatory pattern indicates that SSP may enhance intestinal digestive function by restoring a healthy gut microbiota, thereby indirectly restoring intestinal energy metabolism.

In the comparative correlation analysis between PBT and SSP groups, *Maribacter* and *Scatolibacter* in PBT group showed positive correlations with Ca^2+^-Mg^2+^-ATPase activity. These findings suggest that while pinaverium bromide exerts its direct pharmacological action, it may simultaneously shape an intestine environment that selectively promotes the growth of specific bacteria linked to energy metabolism metrics. However, this distinct microbiota-ATPase association was not detected in SSP group. These findings indicate that SSP likely does not function by specifically enriching a few genera strongly correlated with ATPase. Instead, it appears to work by comprehensively remodeling the gut microecology, thereby restoring the collective function and stability of the microbial community and indirectly improving intestinal energy metabolic homeostasis.

In summary, PBT and SSP exhibit two distinct modes of action. The former, while rapidly alleviating symptoms, shapes a microecology tightly linked to specific microbiota-energy metabolism associations. The latter is dedicated to restoring the overall balance of the microecosystem, with its efficacy not reliant on direct linear relationships between a few key bacterial genera and ATPase. This contrast elucidates, from a microbial ecology perspective, that SSP’s therapeutic strategy focuses more on restoring broad and harmonious interaction networks between the host and the microbial community. Although this study provides new insights into the microecological mechanisms of SSP for treating IBS-D with spleen-kidney yang deficiency, several limitations should be acknowledged. First, the ELISA method used in this study could only detect total ATPase activity in serum and could not discern its cellular origin, particularly whether it stems from intestinal epithelial cells. Second, the animal sample size was relatively small (*n* = 10), utilized only female mice, and did not account for the potential influence of sex differences on the outcomes. Finally, all findings are based on a preclinical animal model, and their clinical translatability requires further validation in human trials. Current research on the mechanism of SSP in treating IBS-D has yielded significant results; however, the specific contributions of its individual herbal components and their interactions require further investigation. Furthermore, existing studies commonly face issues such as small sample sizes, unspecified botanical sources of herbs, and a lack of cross-validation using multiple systemic approaches, which can affect the reliability of the findings. Future research should address these problems and investigate the mechanisms of each component in SSP to provide a more robust scientific basis for its clinical application. In conclusion, while the efficacy of SSP in treating spleen-kidney yang deficiency IBS-D is widely recognized in clinical practice, its precise mechanisms of action warrant further exploration.

## 6 Conclusion

The results of this study demonstrate that SSP effectively alleviates diarrheal symptoms and improves the general condition in an IBS-D model with spleen-kidney yang deficiency. Its therapeutic mechanism is closely associated with the modulation of gut microbiota structure and the restoration of intestinal energy metabolism (reflected by ATPase activity) and digestive function. Correlation analyses further elucidated the intrinsic relationships between intestinal microbiota and host metabolism. In conclusion, SSP affects spleen-kidney yang deficiency IBS-D primarily through its regulation of the “intestinal microbiota-energy metabolism” axis, which represents a key microecological mechanism of action.

## Data Availability

The data presented in this study are publicly available. This data can be found at: https://www.ncbi.nlm.nih.gov/sra, accession PRJNA1177239.

## References

[ref1] Bian L. Q. Huang S. G. Wei W. Wen Y. D. Tang X. D. (2017). Expert consensus on traditional Chinese medicine diagnosis and treatment of irritable bowel syndrome (2024). J. Tradit. Chin. Med. 58, 1614–1620. doi: 10.13288/j.11-2166/r.2024.18.017

[ref2] Chen Z. M. Hu C. J. Pan X. Zhao L. Xiong R. Geng Y. Y. . (2015). Effects of processing psoralea and nutmeg on energy metabolism in rats with spleen-kidney yang deficiency diarrhea. Chin. Tradit. Patent Med. 37, 1298–1301. doi: 10.3969/j.issn.1001-1528.2015.06.030

[ref3] Chen F. Yin Y. T. Zhao H. M. Wang H. Y. Zhong Y. B. Long J. . (2020). Sishen pill treatment of DSS-induced colitis via regulating interaction with inflammatory dendritic cells and gut microbiot. Front. Physiol. 11:801. doi: 10.3389/fphys.2020.00801, PMID: 32754049 PMC7381313

[ref4] Deng N. Xie S. Q. Liu Q. Peng H. Y. Fang L. Y. Shen J. X. . (2025). The intestinal microbiota modulates the visceral sensitivity involved in IBS induced by restraint combined with tail clustering. Front. Cell. Infect. Microbiol. 15:1549617. doi: 10.3389/fcimb.2025.1549617, PMID: 40051709 PMC11882872

[ref5] Deng N. Xie S. Q. Tan Z. J. (2024). Establishment and validation of a mouse model of diarrhea-predominant irritable bowel syndrome with spleen-kidney yang deficiency syndrome. J. Tradit. Chin. Med. 65, 2572–2579. doi: 10.13288/j.11-2166/r.2024.24.011

[ref6] Duan H. J. Hu J. Y. Deng Y. Zou J. Q. Ding W. L. Peng Q. . (2023). Berberine mediates the production of butyrate to ameliorate cerebral ischemia via the gut microbiota in mice. Nutrients 16:9. doi: 10.3390/nu16010009, PMID: 38201839 PMC10781073

[ref7] Fang L. Y. Shen J. X. Wu Y. Tan Z. J. (2025). Involvement of intestinal mucosal microbiota in adenine-induced liver function injury. 3 Biotech 15:6. doi: 10.1007/s13205-024-04180-7, PMID: 39676888 PMC11638458

[ref8] Fogelson K. A. Dorrestein P. C. Zarrinpar A. Knight R. (2023). The gut microbial bile acid modulation and its relevance to digestive health and diseases. Gastroenterology 164, 1069–1085. doi: 10.1053/j.gastro.2023.02.022, PMID: 36841488 PMC10205675

[ref9] Fu Y. Chen Y. Q. Gao W. K. Hu P. Chen L. J. (2023). Clinical efficacy of wen shengu yang decoction combined with moxibustion in patients with diarrhea-predominant irritable bowel syndrome and spleen-kidney yang deficiency syndrome. Chin. Tradit. Patent Med. 45, 90–93. doi: 10.3969/j.issn.1001-1528.2023.01.017

[ref10] Ge W. Zhao H. M. Li Y. Z. Pan Q. H. Liu D. Y. Wang H. Y. . (2022). Mechanism of Sishen pill in regulating colonic energy metabolism in colitis mice. Chin. J. Tradit. Chin. Med. Pharm. 37, 169–173.

[ref11] Guo R. Wei Q. S. Li W. B. Zhu H. Yu G. W. (2019). Research progress on short-chain fatty acids in improving obesity through the gut-brain axis. Clin. Focus. 34, 1148–1152. doi: 10.3969/j.issn.1004-583X.2019.12.020

[ref12] Han B. Y. (2019). Establishment and exploration of biological basis of IBS-D rat model with spleen-kidney yang deficiency syndrome. Beijing: Beijing University of Traditional Chinese Medicine.

[ref13] He Y. S. Tan Z. J. Li D. D. Hui H. Y. (2019). Effect of Bao-he pills on intestinal microorganisms and enzyme activity in mice with dyspepsia. Chin. J. Microecol. 31, 763–767. doi: 10.13381/j.cnki.cjm.201907004

[ref14] Hu X. Y. Guo J. Zhao C. J. Jiang P. Maimai T. Yanyi L. . (2020). The gut microbiota contributes to the development of *Staphylococcus aureus*-induced mastitis in mice. ISME J. 14, 1897–1910. doi: 10.1038/s41396-020-0651-1, PMID: 32341472 PMC7305118

[ref15] Kamiya T. Osaga S. Kubota E. Fukudo S. Motoya S. Murakami K. . (2020). Questionnaire-based survey on epidemiology of functional gastrointestinal disorders and current status of gastrointestinal motility testing in Asian countries. Digestion 102, 73–89. doi: 10.1159/000513292, PMID: 33326975

[ref16] Lacy B. E. Pimentel M. Brenner D. M. Chey W. D. Keefer L. A. Long M. D. . (2021). ACG clinical guideline: Management of Irritable Bowel Syndrome. Am. J. Gastroenterol. 116, 17–44. doi: 10.14309/ajg.0000000000001036, PMID: 33315591

[ref17] Li X. Y. Deng N. Zheng T. Qiao B. Peng M. J. Xiao N. Q. . (2022). Importance of *dendrobium officinale* in improving the adverse effects of high-fat diet on mice associated with intestinal contents microbiota. Front. Nutr. 9:957334. doi: 10.3389/fnut.2022.957334, PMID: 35967811 PMC9365999

[ref18] Li X. Y. Zhu J. Y. Wu Y. Tan Z. J. (2023). Correlation between kidney function and intestinal biological characteristics of adenine and folium sennaeinduced diarrhea model in mice. Turk J Gastroenterol 34, 4–12. doi: 10.5152/tjg.2022.211010, PMID: 35946892 PMC9984907

[ref19] Lin X. Zhang C. Cao K. Li Z. Zhao Z. Li X. . (2023). Dietary sodium butyrate changed intestinal histology and microbiota of rainbow trout (*Oncorhynchus mykiss*), but did not promote growth and nutrient utilization. Aquac. Nutr. 2023, 1–11. doi: 10.1155/2023/3706109, PMID: 36860983 PMC9973217

[ref20] Liu J. X. Wang Y. L. Li Y. Zou D. X. Wang D. F. Ma X. R. . (2019). Experimental study on the effects of sishen pill on gut microbiota in diarrhea-predominant irritable bowel syndrome rats. Acta Pharm. Sin. 54, 670–677. doi: 10.16438/j.0513-4870.2018-0920

[ref21] Liu M. Xue H. Hu Y. L. (2021). Experimental study of the effects of Sishen pill on IBS-D model rats and isolated colon. World Sci. Technol. Mod. Tradit. Chin. Med. 23, 75–80. doi: 10.11842/wst.20200529011

[ref22] Long S. L. Gahan C. G. M. Joyce S. A. (2017). Interactions between gut bacteria and bile in health and disease. Mol. Asp. Med. 56, 54–65. doi: 10.1016/j.mam.2017.06.002, PMID: 28602676

[ref23] Lv Y. C. (2020). Effects of Jianpi Huashi granules on Na+-K+-ATPase activity and the expression of AQPs and Occludin in the colon of IBS-D rats. North China: North China University of Science and Technology.

[ref24] Magalhães D. Cabral J. M. Soares-da-Silva P. Magro F. (2016). Role of epithelial ion transports in inflammatory bowel disease. Am. J. Physiol. Gastrointest. Liver Physiol. 310, G460–G447. doi: 10.1152/ajpgi.00369.2015 26744474

[ref25] Parker B. J. Wearsch P. A. Veloo A. C. M. Rodriguez-Palacios A. (2020). The genus *Alistipes*: gut Bacteria with emerging implications to inflammation, Cancer, and mental health. Front. Immunol. 11:906. doi: 10.3389/fimmu.2020.00906, PMID: 32582143 PMC7296073

[ref26] Petersen C. Bell R. Klag K. A. Lee S. H. Soto R. Ghazaryan A. . (2019). T cell-mediated regulation of the microbiota protects against obesity. Science 365:eaat9351. doi: 10.1126/science.aat9351, PMID: 31346040 PMC7294966

[ref27] Pongkorpsakol P. Yimnual C. Chatsudthipong V. Rukachaisirikul V. Muanprasat C. (2017). Cellular mechanisms underlying the inhibitory effect of flufenamic acid on chloride secretion in human intestinal epithelial cells. J. Pharmacol. Sci. 134, 93–100. doi: 10.1016/j.jphs.2017.05.009, PMID: 28651800

[ref28] Qiao B. Liu J. Deng N. Cai Y. Bian Y. Wu Y. Y. . (2023a). Gut content microbiota dysbiosis and dysregulated lipid metabolism in diarrhea caused by high-fat diet in a fatigued state. Food Funct. 14, 3880–3892. doi: 10.1039/d3fo00378g, PMID: 37038883

[ref29] Qiao B. Liu J. Li D. D. Li X. Y. Liu Y. W. Tan Z. J. (2023b). Comparison of five modeling methods for spleen qi deficiency syndromes based on the theory of diet and overwork injuring the spleen. J Tradit Chin Me. 64, 1149–1156. doi: 10.13288/j.11-2166/r.2023.11.013

[ref30] Sachdeva S. Rawat A. K. Reddy R. S. Puri A. S. (2011). Small intestinal bacterial overgrowth (SIBO) in irritable bowel syndrome: frequency and predictor. J. Gastroenterol. Hepatol. 26 Suppl 3, 135–138. doi: 10.1111/j.1440-1746.2011.06654.x, PMID: 21443727

[ref31] Segata N. Izard J. Waldron L. Gevers D. Miropolsky L. Garrett W. S. . (2011). Metagenomic biomarker discovery and explanation. Genome Biol. 12:R60. doi: 10.1186/gb-2011-12-6-r60, PMID: 21702898 PMC3218848

[ref32] Shahid A. Siddiqui A. J. Musharraf S. G. Liu C. G. Malik S. Syafiuddin A. . (2022). Untargeted metabolomics of the alkaliphilic cyanobacterium *Plectonema terebrans* elucidated novel stress-responsive metabolic modulations. J. Proteome 252:104447. doi: 10.1016/j.jprot.2021.104447, PMID: 34890867

[ref33] Shen X. Guan Z. A. (2022). A study on the action mechanism of Sishen wan on diarrhea irritable bowel syndrome based on network pharmacology and molecular docking. Clin. J. Chin. Med. 14, 7–11. doi: 10.3969/j.issn.1674-7860.2022.22.002

[ref34] Shetty S. Kamble A. Singh H. (2024). Insights into the potential role of plasmids in the versatility of the genus Pantoea. Mol. Biotechnol. 66, 3398–3414. doi: 10.1007/s12033-023-00960-3, PMID: 38007817

[ref35] Singh S. B. Carroll-Portillo A. Lin H. C. (2023). *Desulfovibrio* in the gut: the enemy within? Microorganisms. 11:1172. doi: 10.3390/microorganisms11071772, PMID: 37512944 PMC10383351

[ref36] Streifel A. C. Varley C. D. Ham Y. Sikka M. K. Lewis J. S. (2022). The challenge of antibiotic selection in prosthetic joint infections due to *Corynebacterium striatum*: a case report. BMC Infect. Dis. 22:290. doi: 10.1186/s12879-022-07270-0, PMID: 35346085 PMC8962155

[ref37] Tao J. Kou S. Tang X. Y. (2019). Regulatory effects of ginger on gut microbiota and local immune function in mice. J. Mudanjiang Med. Univ. 40, 15–17. doi: 10.13799/j.cnki.mdjyxyxb.2019.05.005

[ref38] Wang H. Q. Wang S. Y. Wang B. Q. Zhang M. Zheng L. H. (2024). Exploring the effects of acupuncture on IBS through gut microbiota. J. Acupunct. Clin. Stud. 40, 81–86. doi: 10.19917/j.cnki.1005-0779.024098

[ref39] Wu Y. Peng X. X. Li X. Y. Li D. D. Tan Z. J. Yu R. (2022). Sex hormones influence the intestinal microbiota composition in mice. Front. Microbiol. 13:964847. doi: 10.3389/fmicb.2022.964847, PMID: 36386696 PMC9659915

[ref40] Wu Y. Zhang C. Y. Shao H. Q. Luo H. H. Tan Z. J. (2021). Characteristics of intestinal microbiota and enzyme activities in mice fed with lily bulb. 3 Biotech 11:17. doi: 10.1007/s13205-020-02597-4, PMID: 33442516 PMC7778670

[ref41] Xie S. O. Deng N. Fang L. Y. Shen J. X. Tan Z. J. Cai Y. (2024b). TMAO is involved in kidney-yang deficiency syndrome diarrhea by mediating the “gut-kidney axis”. Heliyon 10:e35461. doi: 10.1016/j.heliyon.2024.e35461, PMID: 39170478 PMC11336722

[ref42] Xie S. Q. Fang L. Y. Deng N. Shen J. X. Tan Z. J. Peng X. X. (2024a). Targeting the gut-kidney axis in diarrhea with kidney-yang deficiency syndrome: the role of sishen pills in regulating TMAO-mediated inflammatory response. Med. Sci. Monit. 30:e944185. doi: 10.12659/MSM.944185, PMID: 38898640 PMC11305074

[ref43] Xie J. P. Yuan Y. Tan H. Bai Y. Zheng Q. Y. Mao L. . (2022). The combination of living *Bifidobacterium*, *Lactobacillus*, and *Streptococcus* improves social ranking and relieves anxiety-like behaviors in competitive mice in a social dominance tube test. Brain Behav. 12:e2453. doi: 10.1002/brb3.2453, PMID: 34878231 PMC8785616

[ref44] Zhang Q. Li S. Q. Hu Y. L. Wen N. Wang C. Q. Liu B. B. . (2025). Exploring the mechanism of Sishen pill in improving the intestinal barrier function in IBS-D rats with spleen-kidney yang deficiency based on gut microbiota and SCFAs. Chin J Exp Tradit Chin Med. 31, 80–89. doi: 10.13422/j.cnki.syfjx.20251698

[ref45] Zhang X. W. Mu C. L. Zhu W. Y. (2018). Advances in interactions between gut microbiota and mitochondria. Acta Microbiol Sin. 58, 1908–1915. doi: 10.13343/j.cnki.wsxb.2018019

[ref46] Zhang J. S. Shi X. L. Wang Y. (2024). Exploring causality in the association between gut microbiota and irritable bowel syndrome risk: a large Mendelian randomization study. Aging 16, 7448–7459. doi: 10.18632/aging.205771 38669090 PMC11087118

[ref47] Zhang C. Y. Yao X. Sun G. Yang Y. S. (2019). Close association between abnormal enzyme expression in energy metabolism and diarrhea-predominant irritable bowel syndrome. Chin. Med. J. 132, 135–144. doi: 10.1097/CM9.0000000000000003 30614852 PMC6365280

[ref48] Zhang X. Y. Zhao H. M. Liu Y. Lu X. Y. Li Y. Z. Pan Q. H. . (2021). Sishen pill maintained colonic mucosal barrier integrity to treat ulcerative colitis via rho/ROCK signaling pathway. Evid. Based Complement. Alternat. Med. 2021:5536679. doi: 10.1155/2021/5536679, PMID: 34925530 PMC8677397

[ref49] Zhou M. S. Li X. Y. Wang X. H. Deng N. Cai Y. Tan Z. J. (2024). The dysfunction in intestinal microorganisms and enzyme activity significantly contributors to diarrhea with kidney-yang deficiency syndrome. Front. Microbiol. 14:1324938. doi: 10.3389/fmicb.2023.1324938, PMID: 38264481 PMC10803573

[ref50] Zhou M. S. Li X. Y. Xiao N. Q. Tan Z. J. (2025). One mechanism of Sishen pill on diarrhea with kidney Yang deficiency syndrome: influencing metabolic function by intestinal microorganisms and enzyme activity mediates the gut-kidney axis. Front. Cell. Infect. Microbiol. 15:1620789. doi: 10.3389/fcimb.2025.1620789, PMID: 40895300 PMC12390983

[ref51] Zhu H. Y. (2014). Mechanism of liver-smoothing and spleen-strengthening method on cellular energy metabolism in irritable bowel syndrome rat model. Acta Chin. Med. Pharmacol. 42, 36–38. doi: 10.19664/j.cnki.1002-2392.2014.02.013

[ref52] Zhu J. Y. Li X. Y. Deng N. Peng X. X. Tan Z. J. (2022). Diarrhea with deficiency kidney-yang syndrome caused by adenine combined with folium senna was associated with gut mucosal microbiota. Front. Microbiol. 13:1007609. doi: 10.3389/fmicb.2022.1007609, PMID: 36304943 PMC9593090

[ref53] Zhu J. Y. Li X. Y. Deng N. Zhou K. Qiao B. Li D. D. . (2023). Intestinal mucosal flora of the intestine-kidney remediation process of diarrhea with deficiency kidney-yang syndrome in Sishen pill treatment: association with interactions between *Lactobacillus johnsonii*, Ca^2+^-Mg^2+^-ATP-ase, and Na^+^-K^+^-ATP-ase. Heliyon 9:e16166. doi: 10.1016/j.heliyon.2023.e16166, PMID: 37215812 PMC10199185

