# Correction: Sishen Pill treats spleen-kidney yang deficiency IBS-D by influencing ATPase activity and intestinal microbiota

**DOI:** 10.3389/fmicb.2026.1919046

**Published:** 2026-07-21

**Authors:** Qi Long, Liwen Li, Zhoujin Tan, Na Deng

**Affiliations:** 1 School of Traditional Chinese Medicine, Hunan University of Chinese Medicine, Changsha, China; 2 Hunan Key Laboratory of Traditional Chinese Medicine Prescription and Syndromes Translational Medicine, Changsha, China

**Keywords:** Sishen Pill, intestinal microbiota, spleen-kidney yang deficiency IBS-D, ATPase activity, intestinal digestive enzyme activity

In the published article, there was a mistake in Figure 1. The flowchart incorrectly displayed the durations of the adaptive feeding period and the *Folium senna* administration period.

**Figure 1:**
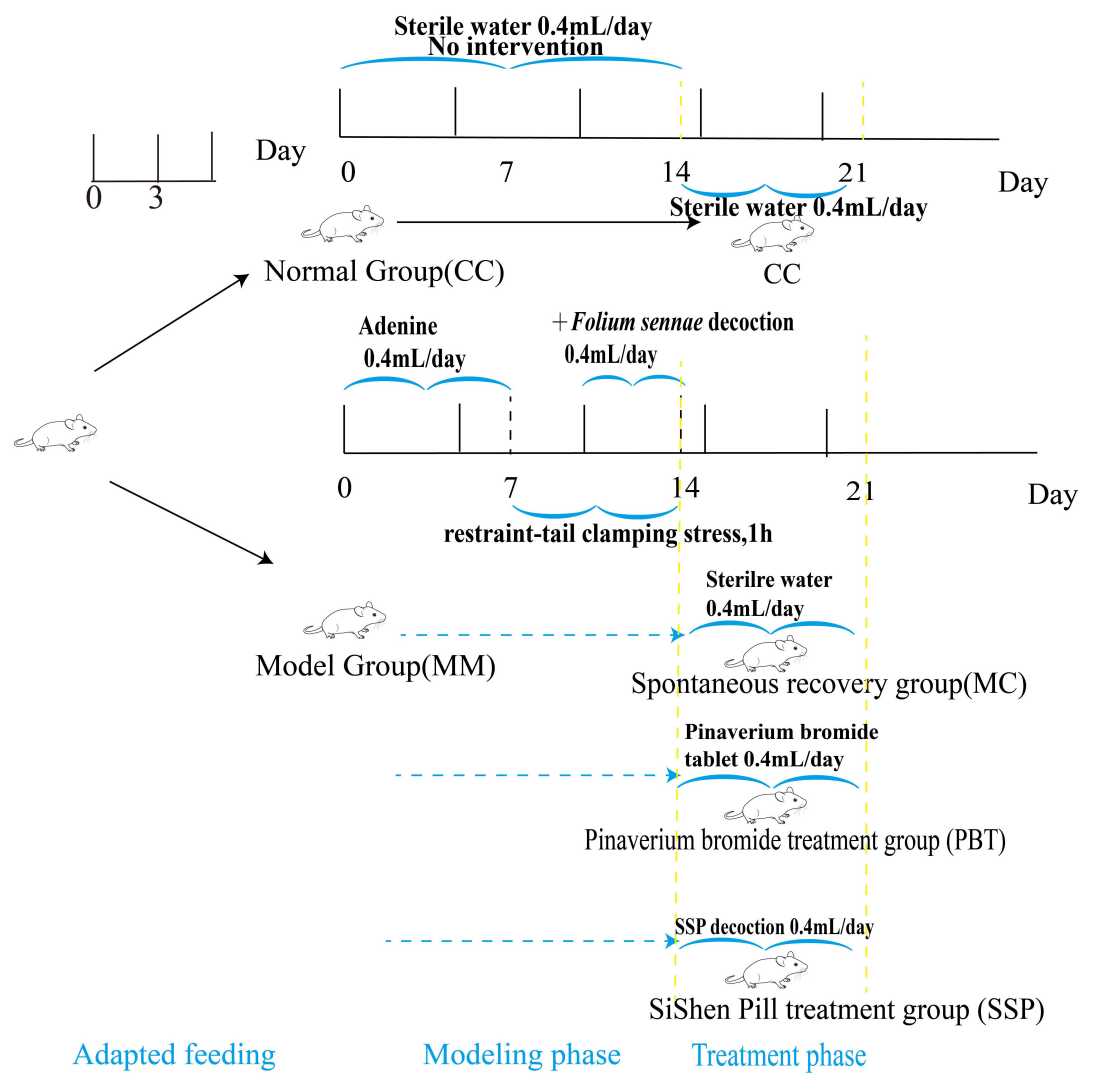
Experimental design and general conditions of the animals. CC, normal group; MC, spontaneous recovery group; PBT, pinaverium bromide tablet group; SSP, Sishen Pill group.

The correct durations have been revised in the updated figure. The corrected Figure 1 appears above.

In the published article, there were two errors in **3 Methods**, *3.1 Grouping and modeling methods*, Paragraph 1, page 3.

The duration of *Folium senna* administration was incorrectly stated as “7 days” and should be corrected to “5 days.”

Figure 1: The adaptive feeding duration label has been corrected from “6 days” to “3 days” in the experimental timeline illustration.

**The original sentence was**: After 7 days of adaptive feeding, 40 mice were randomly divided into two groups: a normal group (CC) (10 mice) and a model group (MM) (30 mice). The IBS-D mouse model with spleen-kidney yang deficiency was induced by administering adenine + *Folium sennae* decoction combined with restraint-tail clamping stress, as described in a previous study (Deng et al., 2024). One week prior to modeling, the model group was gavaged with an adenine suspension [50 mg/(kg·d), 0.4 mL per mouse] for 14 consecutive days. Beginning on the 8th day of modeling, the limbs of the model group mice were restrained in centrifuge tubes, and the distal third of their tails were clamped for 1 h daily for 7 days. In the afternoon, they were gavaged with *Folium sennae* decoction [10 g/(kg·d), 0.4 mL/mouse, once daily, for 7 days], while CC received sterile water at the same dosage.

**The corrected sentence should read:** After 3 days of adaptive feeding, 40 mice were randomly divided into two groups: a normal group (CC; 10 mice) and a model group (MM; 30 mice). The IBS-D mouse model with spleen-kidney yang deficiency was induced by administering adenine + *Folium sennae* decoction combined with restraint-tail clamping stress, as described in a previous study (Deng et al., 2024). One week prior to modeling, the model group was gavaged with an adenine suspension [50 mg/(kg·d), 0.4 ml per mouse] for 14 consecutive days. Beginning on the 8th day of modeling, the limbs of the model group mice were restrained in centrifuge tubes, and the distal third of their tails were clamped for 1 h daily for 7 days. Starting from day 10, model group mice were gavaged with *Folium sennae* decoction in the afternoon [10 g/(kg·d), 0.4 ml/mouse, once a day, for 5 consecutive days], while CC received sterile water at the same dosage.

The original version of this article has been updated.

